# Predictors of early child development for screening pregnant women most in need of support in Brazil

**DOI:** 10.7189/jogh.14.04143

**Published:** 2024-08-23

**Authors:** Eduardo Viegas da Silva, Fernando Pires Hartwig, Thiago Melo Santos, Aisha Yousafzai, Iná S Santos, Aluísio J D Barros, Andréa Dâmaso Bertoldi, Mariângela Freitas da Silveira, Alicia Matijasevich, Marlos Rodrigues Domingues, Joseph Murray

**Affiliations:** 1 Postgraduate Programme in Epidemiology, Federal University of Pelotas, Pelotas, Rio Grande do Sul, Brazil; 2 Human Development and Violence Research Centre, Federal University of Pelotas, Pelotas, Rio Grande do Sul, Brazil; 3 International Center for Equity in Health, Federal University of Pelotas, Pelotas, Rio Grande do Sul, Brazil; 4 Global Health and Population Department, Harvard School of Public Health, Boston, USA; 5 Departamento de Medicina Preventiva, Faculdade de Medicina FMUSP, Universidade de São Paulo, São Paulo, Brazil; 6 Postgraduate Programme in Physical Education, Federal University of Pelotas, Pelotas, Rio Grande do Sul, Brazil

## Abstract

**Background:**

Home visiting programmes can support child development and reduce inequalities, but failure to identify the most vulnerable families can undermine such efforts. We examined whether there are strong predictors of poor child development that could be used to screen pregnant women in primary health care settings to target early interventions in a Brazilian population. Considering selected predictors, we assessed coverage and focus of a large-scale home visiting programme named *Primeira Infância Melhor* (*PIM*)

**Methods:**

We undertook a prospective cohort study on 3603 children whom we followed from gestation to age four years. We then used 27 potential socioeconomic, psychosocial, and clinical risk factors measurable during pregnancy to predict child development, which was assessed by the Battelle Developmental Inventory (BDI) at the age of four years. We compared the results from a Bonferroni-adjusted conditional inference tree with exploratory linear regression and principal component analysis (PCA), and we conducted external validation using data from a second cohort from the same population. Lastly, we assessed *PIM* coverage and focus by linking 2015 cohort data with *PIM* databases.

**Results:**

The decision tree analyses identified maternal schooling as the most important variable for predicting BDI, followed by paternal schooling. Based on these variables, a group of 214 children who had the lowest mean BDI (BDI = −0.48; 95% confidence interval (CI) = −0.63, −0.33) was defined by mothers with ≤5 years and fathers with ≤4 years of schooling. Maternal and paternal schooling were also the strongest predictors in the exploratory analysis using regression and PCA, showing linear associations with the outcome. However, their capacity to explain outcome variance was low, with an adjusted R^2^ of 5.3% and an area under the receiver operating characteristic curve of 0.62 (95% CI = 0.60, 0.64). External validation showed consistent results. We also provided an online screening tool using parental schooling data to support programme’s targeting. *PIM* coverage during pregnancy was low, but the focus was adequate, especially among families with longer enrolment, indicating families most in need received higher dosage.

**Conclusions:**

Information on maternal and paternal schooling can improve the focus of home visiting programmes if used for initial population-level screening of pregnant women in Brazil. However, enrolment decisions require complementary information on parental resources and direct interactions with families to jointly decide on inclusion.

Adequate early child development (ECD) provides a strong foundation for physical and mental health, educational attainment, and income across the lifespan [1–4]. However, worldwide, hundreds of millions of children under the age of five years are at risk of not reaching their developmental potential [5,6]. In early childhood, particularly the first 1000 days of life, greater brain plasticity and sensitivity to environmental stimuli provide an important window of opportunity for interventions to support development and improve later outcomes [7].

Parental programming for nurturing care occurs in part during pregnancy [8–10]. Consequently, programmes starting during this period can improve parental knowledge and attitudes about childrearing at birth, potentially impacting responsive caregiving and parent-child attachment [10–12]. From an implementation perspective, pregnancy can be an optimal time to establish engaging relationships between an expectant mother and service providers, while more time can be invested in maternal self-care.

Systematic reviews have indicated that home visiting programmes can have moderate to large positive effects on ECD (e.g. effect sizes between 0.38 to 0.48 standard deviations (SDs) for cognitive development and 0.28 to 0.47 for language development) [13–15]. However, implementation features are crucial to positive impact, requiring an understanding of issues in each context to scale up effective interventions [16]. Greater benefits have been observed with disadvantaged populations [8,13–15,17]. Therefore, to reduce inequalities in ECD, programmes should aim to ensure the most vulnerable families are included in their scope, particularly if resources are limited. Many large-scale home visiting programmes use one or two broad eligibility criteria, such as teenage mothers, first-time mothers, families below a poverty line, or being enrolled in social security programmes [18–22]. Some other programmes have a long list of criteria to consider families for enrolment, but lack an objective tool to determine eligibility [23]. To our knowledge, no programme has established the predictive validity of its eligibility criteria for ECD outcomes. Predicting future child developmental progress is challenging, especially before birth. Several predictive risk models for child development have shown good predictive capacity [24–30], but only one study used information restricted to the period of pregnancy. Testing six potential predictors in total, the Avon cohort study (England) found an area under the receiver operating characteristic curve (AUC) smaller than 0.70, suggesting poor discriminatory power [18].

In the southernmost state of Brazil, a large-scale early home visiting programme called *Primeira Infância Melhor* (*PIM*) (Eng. Better Early Childhood) has been implemented as a public policy since 2003. Through weekly home visits (45–60-minute) by trained non-professionals, *PIM* aims to enhance responsive caregiver-child interactions through engagement in age-appropriate play activities, along with provision of information for supporting nurturing care. It has served more than 60 000 pregnant women and 250 000 children to date [31]. While the programme considers a broad list of indicators in selecting families for enrolment [23], it utilises no objective tool. Visitors and supervisors enrol families based on field experience and referrals from social services. However, limitations in resources restrict the application of *PIM* (34% of children receiving *PIM* in one city were subsequently withdrawn due to lack of a visitor [32]), so successful targeting of vulnerable families is critical. Inspired by the PIM programme, Brazil implemented the largest home visiting programme in the world in 2016, naming it *Programa Criança Feliz* (*PCF*) (Eng. Happy Child Programme). Families are eligible for *PCF* if they registered in a federal system for a cash transfer programme and other social benefits [21]. However, this broad criterion identifies many more families than the programme can effectively serve [17]. Significantly, both *PIM* and *PCF* aim to enrol most families during pregnancy, but in practice, most are enrolled after birth [32,33]. Thus, better targeting of pregnant women using simple screening information could improve the effectiveness of both programmes and help reduce inequalities in ECD, given that both programmes need to strengthen delivery strategies [34].

The proportion of adults without completed primary school education is 42% in Pelotas city and 45% in Brazil [35], and fewer than 1/3 of municipalities across the country have a high score on indices of nurturing care environments [36]. On the other hand, Brazil’s universal health system has high primary health care (PHC) coverage in poorer communities [37], with a demonstrated potential to effectively integrate it with home visiting programmes to promote ECD, particularly during prenatal care [38].

No prior study in low- and middle-income countries (LMICs) has examined a prediction model of ECD using only variables measurable during pregnancy, and only one did so in a high-income country [18]. Our study builds upon prior research that identified strong predictors [18,24–30] by adding new, previously untested ones. It also provides novel information about population-level coverage and focus of a large-scale home visiting programme (*PIM*) to promote ECD in Brazil, to support better targeting of such programmes. We aimed to examine whether there are indicators available during pregnancy that are strongly predictive of childhood development at age four years, to enable screening of pregnant women in PHC settings for early inclusion in programmes to support ECD. We further sought to use these predictors to assess the extent of coverage and focus of *PIM* initiated during pregnancy, among children born in 2015 in Pelotas city in Southern Brazil.

## METHODS

### Design and participants

All children delivered in hospitals in Pelotas (a city with around 340 000 inhabitants) between 1 January and 31 December 2015 whose mother lived in the urban area of the city were eligible for the 2015 Pelotas Birth Cohort Study. From the 4333 eligible live births, 3199 (73.8%) expectant mothers were interviewed in prenatal period, 4275 (98.7%) children were assessed at birth, and subsequently invited to complete follow-up assessments at 3 (97.2%), 12 (95.3%), 24 (95.3%) months, and 4 years (95.3%). Therefore, on follow-up at 4 years, our overall response rate was 95.3%, including 4010 participants who were assessed and 67 participants who were identified as having died. The 24-month and the 4-year follow-ups were conducted in a university research centre; prior visits, in turn, occurred in children’s homes. Further information about the 2015 Pelotas Birth Cohort is available elsewhere [39].

To identify children receiving *PIM*, we linked primary data from the cohort and secondary data from the *PIM* information system based on municipality (Pelotas), child's date of birth, child's name, and mother's name [32]. We also extracted information on timing of enrolment in *PIM* (before or after birth) and duration of the intervention (less than 12 months or 12 months or more) to help examine coverage and focus of the programme.

### Potential predictors

**Figure 1:**
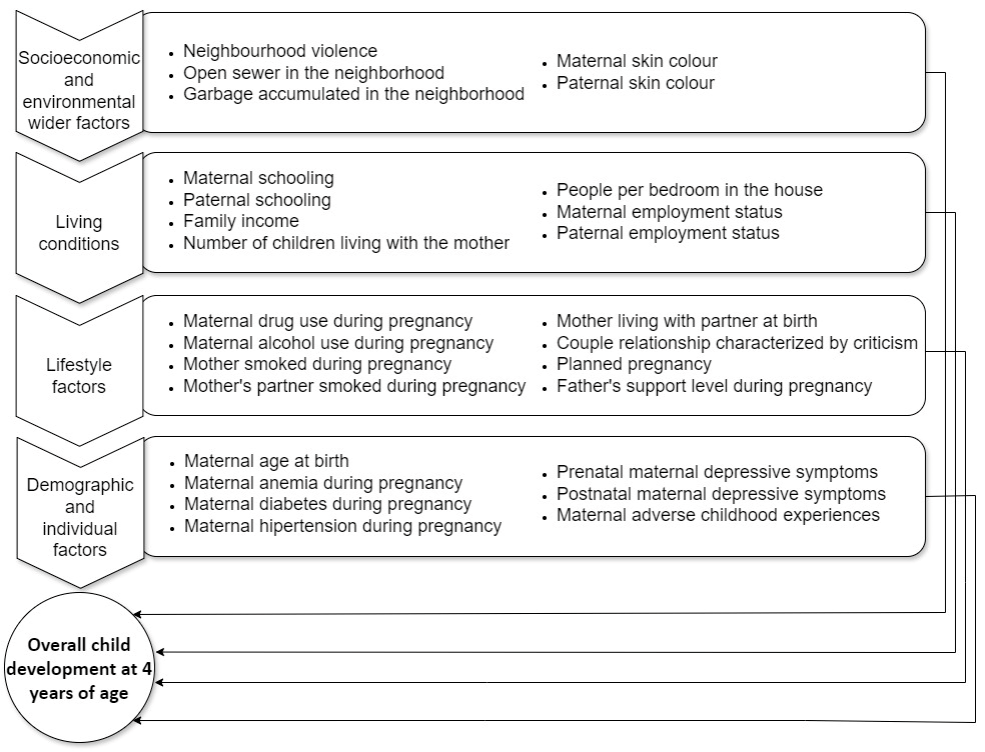
Theoretical model of 27 potential predictors of overall child development at four years of age.

To select a subset of the strongest predictors for use as risk indicators without encountering necessarily aetiological effects, we examined a broad set of potential predictors that are feasibly measurable in Brazilian PHC settings during prenatal care. Specifically, we included all variables identified as strong predictors in prior studies testing predictive models of ECD [18,24–30], except for two: sex of the child (not considered a potential selection criterion for an intervention to promote ECD) and maternal intelligence quotient (IQ) (not assessed in the 2015 Pelotas Birth Cohort). We further included previously untested predictors that were part of the social determinants of health, according to the Dahlgren-Whitehead theoretical model [40]. We conceptualised the final set of 27 predictors included as socioeconomic and broad environmental risks (n = 5), living conditions (n = 7), lifestyle factors (n = 8), and demographic and individual factors (n = 7) (**Figure 1**). Measurement details and operationalisations are described in Box S1 in the **Online Supplementary Document**.

### Outcome

Child development was measured at age four years using the screening version of Battelle’s Developmental Inventory (BDI) [41] (**Online Supplementary Document**). The BDI scores at this age have good validity for predicting later development [42]. The BDI itself had previously been translated to Brazilian Portuguese and was adapted from 96 items to a reduced 66-item instrument (using all items for each age level from birth to 4–5 years of age, but excluding items for older ages). Thus, the total development score can range from 0 to 132 [43]. Trained interviewers supervised by senior psychologists applied the BDI. Quality control for 200 randomly selected children found strong agreement between scores based on coding by senior psychologists and from the original interviewers’ coding. The total scores in the 2015 Pelotas Birth Cohort ranged from 36 to 131 (mean (x̄) = 113.4; SD = 8.8) and had an approximately normal distribution (Figure S1 in the **Online Supplementary Document**). We excluded three children due to severe conditions (BDI<50). We used the score as a continuous outcome to retain maximum information and standardised it based on its distribution in the study sample to improve the interpretation of the differences between subgroups generated by the decision tree in terms of population distribution.

### Statistical analysis

For our descriptive analysis, we presented continuous variables using means and SD and categorical and binary ones using numbers and proportions. We also described the number of individuals with missing data for each variable.

We then used a decision tree approach to identify subgroups of children with similar child development scores at age four years based on information that could potentially be collected during gestation in PHC settings. Decision trees are useful for examining a large set of predictors, as they are flexible and can explore combinations of predictors and nonlinear relationships without testing all combinations. They perform a binary recursive search, meaning that they divide the sample into two smaller subgroups (hence binary) and then continue to divide them into even smaller subgroups (hence recursive) until a stopping rule is triggered [44–46]. A potential limitation of this technique is overfitting the data, which is a special concern when using a relatively large sample (as there is enough statistical power to detect even minor predictive gains) and a large number of predictors (which increases the risk of type I error). To mitigate this, we used the conditional inference tree (Ctree) approach, whereby for the first division, the tree chooses the predictor most strongly associated with the outcome in the sample. The split point (cutoff point) is established to maximise a test statistic that is selected given the types of predictors and outcomes included in the analyses. A formal hypothesis test is performed, and further divisions of the sub-samples stop when the Bonferroni-adjusted *P*-value is larger than 5% [47,48]. We used all standard parameters of the ‘partykit’ package in R, version 4.1.0 (R Core Team, Vienna, Austria), except for the minimum number of children in any subgroup, which we set at 50, and the number of possible surrogates for a predictor with missing data, which we set at 5. A more detailed description of the method is presented in the **Online Supplementary Document** and elsewhere [48].

For the conditional inference tree analysis, we first fitted a linear regression model using child sex and age in months at 4-year follow-up to predict standardised BDI and used residuals as the outcome to generate a decision tree adjusted for those two covariates. We did not consider sex as a potential criterion for selecting children to receive an intervention to promote ECD. We obtained an adjusted R^2^ of a polytomous variable representing the decision tree terminal groups predicting the outcome as a measure of how well outcome variance was explained. In another set of analyses, we took the predictors selected by the decision tree, formally investigated the linearity of their associations with standardised BDI, and afterwards included them in a linear regression model predicting standardised BDI; we then stored the predicted values as a score of childhood development vulnerability. Subsequently, we estimated the AUC and identified the cutoff point of that predicted score that maximised sensitivity and specificity values. For this last accuracy analysis, we dichotomised the outcome BDI at the 10th percentile of the whole cohort; this identified children whose developmental score did not surpass that expected of children aged 30 months according to the BDI instrument’s norms.

In a sensitivity analysis for the decision tree, we excluded 142 children who had been enrolled in the *PIM* intervention during pregnancy, given we had previously identified the effects of the *PIM* programme on childhood development in that subgroup [32].

Considering that the results of the main decision tree analysis did not reveal complex combinations of predictors, we evaluated whether they were consistent with two exploratory analyses using more traditional methods. First, we ran a linear regression model to verify if the predictors selected by the decision tree would remain in the final model and have the strongest associations with the outcome. We included 21 potential predictors of childhood development that were considered to be key predictors in the literature, plus products among 11 of them (Box S2 in the **Online Supplementary Document**). To reduce losses due to missing data, we did not include four predictors measured prenatally with 25% missing (garbage accumulated in the neighbourhood; open sewer in the neighbourhood; maternal drug use during pregnancy; and prenatal maternal depressive symptoms) and we excluded two predictors measured postpartum answered only by mothers living with a partner with 19% missing (couple relationship characterised by criticism and mother's partner smokes at perinatal). In addition, we recategorised five predictors for these analyses: maternal age as <20/≥20 years; maternal skin colour and paternal skin colour as white/non-white; maternal postnatal depression symptoms as low (0–9)/moderate (10–12)/significant (≥13); and family income in quintiles. We applied backward selection in the analyses, so the final model included predictors with *P* < 0.05. We re-entered all excluded covariates into the final model to ensure no strong predictor was left out. We kept children’s sex and age in months at the 4-year follow-up in the model to estimate more realistic coefficients for predictors of interest.

The second exploratory analysis was motivated by the moderate to high correlations we observed between a few of the predictors. To verify the dimensional representation of the data and identify predictors with the highest eigenvalues, we ran a principal component analysis (PCA) of a correlation matrix including 11 potential predictors (the same set for which products were included in the linear regression exploratory analysis). After we inspected the scree plot, we included principal components with eigenvalues >1 in a linear regression model predicting standardised BDI, along with children’s sex and age in months at the 4-year follow-up.

The same BDI instrument was applied at the age of four years in the 2004 Pelotas Birth Cohort Study [49], which has the same methodology (11 years earlier) as the 2015 Pelotas cohort. In the 2004 Pelotas Cohort, total scores had ranged from 02 to 132 (x̄ = 118.3; SD = 8.5). Nine children of the 2004 cohort were excluded due to severe conditions (BDI<50). The partition rules of the decision tree generated in the 2015 Pelotas cohort had been applied in the 2004 Pelotas cohort for external validation. Residuals from linear regression in which sex and age of the child predicted standardised BDI in the 2004 cohort were used as the outcome. We described the outcome distribution within each decision tree terminal group and obtained an adjusted R^2^ of a polytomous variable representing terminal groups. We assessed the discriminatory power by AUC and sensitivity and specificity values.

We then examined the extent of enrolment in *PIM* during pregnancy across children with different predicted scores of development vulnerability at the age of 4 years. We measured coverage as the proportion of children with low predicted BDI who actually received *PIM* starting in pregnancy, and focus as the proportion of children receiving *PIM* from pregnancy with low predicted BDI. We also examined focus according to the duration of the intervention (proportion participating 12 months or more vs less than 12 months).

Finally, after we conducted all analyses of predictors measured during pregnancy, we performed additional post-hoc analyses to examine if two recognised perinatal predictors of child development (gestational age and birth weight) could help improve the identification of children who would benefit from enrolling in the programme soon after birth [1]. To examine this question, we ran the main analysis (decision tree) and the two exploratory analyses (linear regression and PCA) adding these two perinatal predictors to the model.

We conducted the decision tree analysis, including external validation, in R, version 4.1.0, and all other analyses in Stata, version 15.1 (StataCorp LLC, College Station, TX, USA). We followed the STROBE reporting guidelines in presenting our findings [50].

## RESULTS

BDI was collected for 3607 out of 4275 children in the 2015 Pelotas Birth Cohort at the age of four years. We excluded three children due to severe conditions (BDI<50) and one child due to missing data for age at four-year follow-up, resulting in 3603 children in the decision tree analysis (84% of the whole cohort; 86% of those still alive). There was little difference between the whole cohort and the analytic sample (e.g. identical distributions of maternal schooling and maternal depressive symptoms) (Table S1 in the **Online Supplementary Document**).

The unadjusted analyses showed that 20 predictors had a statistically significant association with BDI. Seven, in turn, were not strongly associated (*P* > 0.05), including garbage accumulated in the neighbourhood; open sewer in the neighbourhood; maternal drug use during pregnancy; maternal alcohol use during pregnancy; couple relationship characterised by criticism; maternal diabetes during pregnancy; and maternal anaemia during pregnancy (Table S2 in the **Online Supplementary Document**). Nevertheless, we included all 27 potential predictors measurable during pregnancy in the decision tree analysis to explore possible relevant interactions.

### Decision tree analysis of predictors of child development

**Figure 2:**
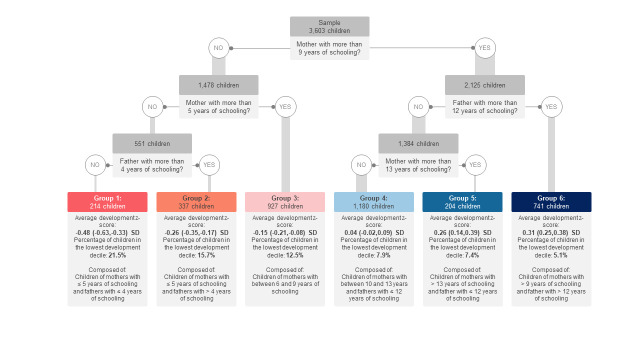
Conditional inference tree of childhood development at age four years in 2015 Pelotas Birth Cohort (n = 3603).

**Figure 3:**
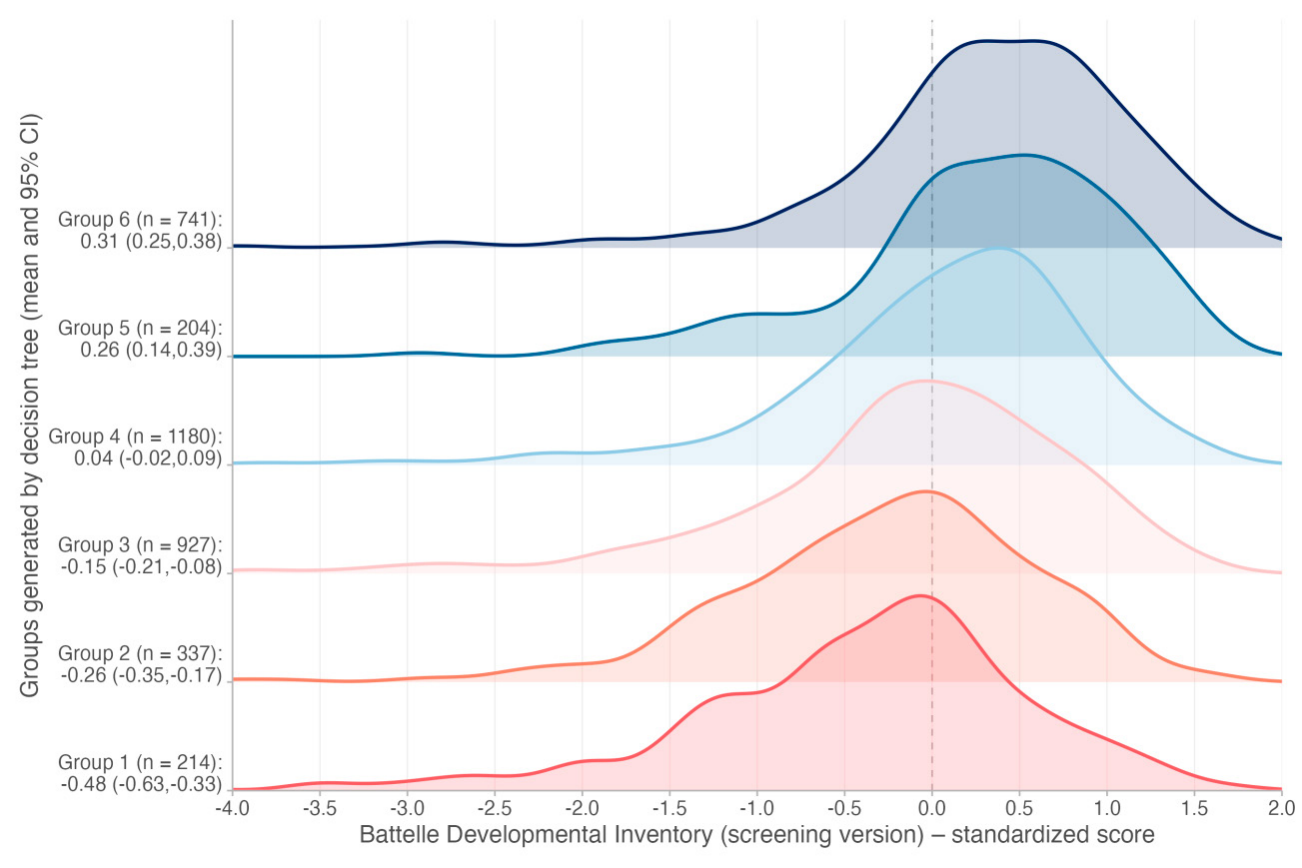
Density distributions of standardised Battelle Developmental Inventory (screening version) at age four years across the six terminal groups generated by the decision tree (n = 3603).

The decision tree automatically selected two predictors of child development: maternal and paternal schooling. First, it split the sample between children whose mothers had ≤9 vs >9 years of schooling. Then, maternal and paternal schooling were used for further ramifications until the tree ended in six groups defined by these two variables. The group with the lowest standardised BDI mean (BDI = −0.48; 95% CI = −0.63, −0.33) comprised 214 children of mothers with ≤5 years of schooling and fathers with ≤4 years of schooling. In the opposite branch, the group with the highest standardised BDI mean (BDI = 0.31; 95% CI = 0.25, 0.38) had 741 children of mothers with >9 years of schooling and fathers with >12 years of schooling (**Figure 2**). This represented a mean difference of 0.79 SD between those two extremes groups. We observed that the density distributions of the outcome were different among terminal groups, although with substantial overlap (**Figure 3**). The six terminal groups explained a small proportion of outcome variance (adjusted R^2^ = 5.3%). Sensitivity analysis (excluding 142 children who had been enrolled in *PIM* during pregnancy) generated a tree selecting the same predictors in the same order – maternal schooling first, followed by paternal schooling.

### Exploratory, alternative models predicting child development

In the first exploratory analysis, we used linear regression to predict child development. The final regression model included five predictors (alongside covariates): maternal schooling (*P* < 0.001); paternal schooling (*P* = 0.001); family income (*P* = 0.015); the product of maternal skin colour × maternal depressive symptoms (*P* = 0.015); and the product of alcohol use in pregnancy × maternal adverse childhood experiences (*P* = 0.037) (**Table 1**). The adjusted R^2^ for those five predictors of interest was low (5.7%). The results did not change after we ran the same regression process after excluding children enrolled in the *PIM* programme during pregnancy.

**Table 1:** Final adjusted linear regression model including predictors strongly associated (*P* ≤ 0.05) with standardised Battelle Developmental Inventory (screening version) at age four years in 2015 Pelotas Birth Cohort (n = 3282)

Predictor	Coefficient (95% CI)	*P*-value*
Maternal schooling in years	0.03 (0.02, 0.04)	<0.001
Paternal schooling in years	0.02 (0.01, 0.03)	0.001
Family income (minimum wage quintile)		0.015
*≤1.0*	0	
*1.1 to 3.0*	0.09 (−0.02, 0.19)	
*3.1 to 6.0*	0.13 (0.01, 0.25)	
*6.1 to 10.0*	0.29 (0.12, 0.45)	
*>10.0*	0.13 (−0.06, 0.31)	
Maternal skin colour × maternal depressive symptoms	−0.06 (−0.10, −0.01)	0.015
Alcohol in pregnancy × maternal adverse childhood experiences	−0.05 (−0.09, −0.00)	0.037

CI – confidence interval

*Wald test.

The second exploratory analysis used PCA. The Kaiser-Meyer-Olkin statistic was acceptable (0.76). The first principal component explained 27% of the 11 predictors’ variance. Representative predictors (eigenvectors >0.30) were maternal schooling (0.49), paternal schooling (0.46) and family income (0.43) (Table S3 in the **Online Supplementary Document**). In total, four principal components had eigenvalues >1 (Figure S2 in the **Online Supplementary Document**). Only the first principal component strongly predicted BDI (**Table 2**), and the adjusted R^2^ for the four principal components was low (5.3%). We tested the same PCA process excluding children enrolled in *PIM* during pregnancy, and the results did not change.

**Table 2:** Linear regression model including four principal components (with eigenvalues >1) predicting standardised Battelle Developmental Inventory (screening version) at age four years in 2015 Pelotas Birth Cohort (n = 3282)

Predictor	Coefficient (95% CI)	*P*-value*
Principal component 1	0.13 (0.11, 0.14)	<0.001
Principal component 2	0.01 (−0.02, 0.04)	0.466
Principal component 3	0.02 (−0.01, 0.05)	0.283
Principal component 4	0.02 (−0.01, 0.05)	0.258

CI – confidence interval

*Wald test.

**Figure 4:**
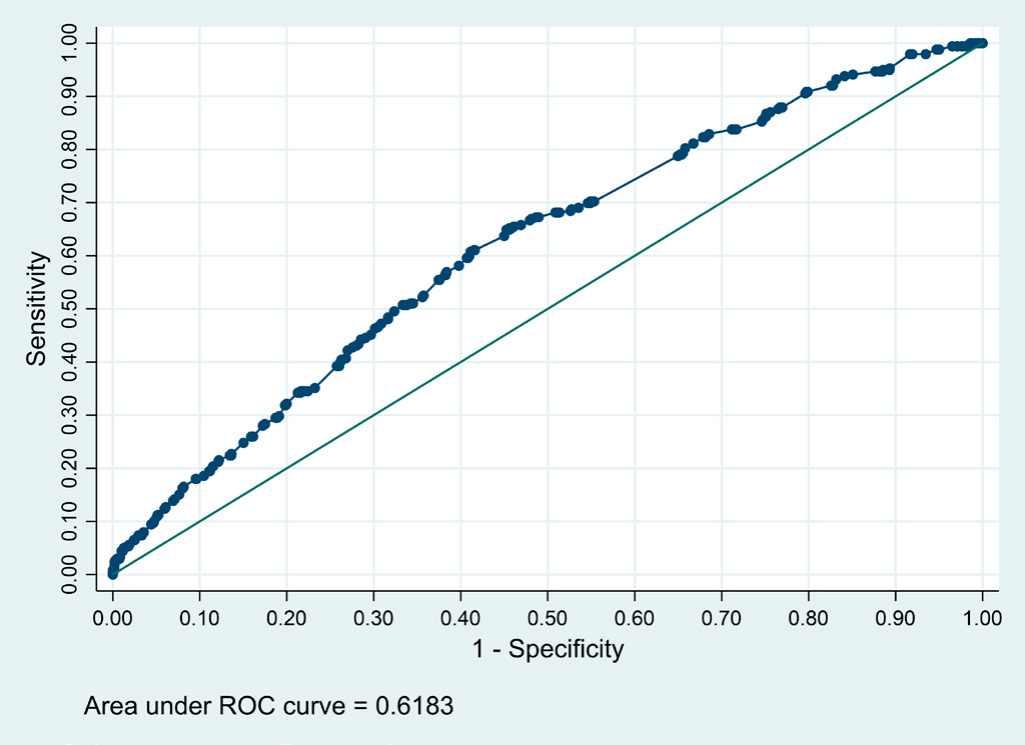
Area under the receiver operating characteristic curve (AUC) for predicted score of childhood development vulnerability (based on maternal and paternal schooling), predicting the outcome low childhood development (below 10th percentile of BDI in the whole cohort) at age four years in 2015 Pelotas Birth Cohort.

Given similar adjusted R^2^ statistics estimated from decision tree analysis (5.3%), linear regression (5.7%) and PCA (5.3%), and the aim to provide a predictive model for real-life screening of pregnant women at the population level in PHC, for simplicity, we used predictors selected by the decision tree in subsequent analyses of discriminatory power and *PIM* programme coverage and focus. We formally investigated the relationships of maternal schooling and paternal schooling with standardised BDI scores through Stata’s standard fractional polynomial feature and found no strong evidence for nonlinearity (Figures S3–4 and Table S4 in the **Online Supplementary Document**). When dealing with linear relationships, the cutoff points selected by the decision trees tend to be quite arbitrary. Therefore, we did not use the decision tree’s terminal groups that were based on those cutoff points. We used linear regression with maternal schooling and paternal schooling to predict standardised BDI and used the predicted values from these models as a score of child developmental vulnerability. Assessing the capacity of this predicted score to discriminate children below the 10th percentile of BDI in the whole 2015 cohort, the AUC was small (AUC = 0.62; 95% CI = 0.60, 0.64) (**Figure 4**) and the cutoff point that maximised accuracy (score equal to or less than −0.02) provided a sensitivity value of 0.61 (95% CI = 0.55, 0.66) and a specificity value of 0.59 (95% CI = 0.57, 0.61) (Table S5 in the **Online Supplementary Document**). Excluding 142 children who received *PIM* during pregnancy, AUC, sensitivity, and specificity did not change.

### External validation of the predictive models in a second cohort

We began the external validation with 2004 Pelotas cohort data (n = 3787) by creating six groups of children defined by the values of maternal and paternal schooling selected by the 2015 Pelotas cohort’s decision tree, after which we examined their BDI scores. We observed that mean BDI scores were higher across all groups in the 2004 cohort than in their respective groups in the 2015 cohort. This was possible, despite both cohorts having mean standardised BDI scores of 0.0, because the size of groups with lower parental education (and low BDI) was larger in the 2004 cohort than in the 2015 cohort. In the 2004 cohort, the leftmost group of children (whose mothers had ≤5 years of schooling and fathers had ≤4 years of schooling; n = 372, 9% of the cohort) had a mean standardized BDI of −0.36 (95% = CI −0.48, −0.24), compared to −0.48 (95% CI = −0.63, −0.33) in the 2015 cohort (n = 214, 5% of the entire cohort). We observed a mean difference of 0.86 SD of BDI scores between the two extreme groups characterised by the highest vs lowest levels of parental schooling in the 2004 cohort (Figure S5 in the **Online Supplementary Document**). The adjusted R^2^ for the six terminal groups predicting standardised BDI was 6.7% in the 2004 cohort. Formally investigating the relationships of maternal and paternal schooling with standardised BDI scores in the 2004 cohort, we found no strong evidence for nonlinearity (Figures S6–7, Table S6 in the **Online Supplementary Document**). The AUC of the predicted BDI score (based on maternal and paternal schooling) to discriminate children below the 10th percentile of BDI was 0.66 (95% CI = 0.64, 0.68) in the 2004 cohort. Using the cutoff point previously derived from the 2015 cohort (score equal to or less than −0.02) predicted low BDI in the 2004 cohort, with a sensitivity of 0.73 (95% CI = 0.68, 0.78) and a specificity of 0.53 (95% CI = 0.52, 0.55).

### Coverage and focus of *PIM*

**Figure 5:**
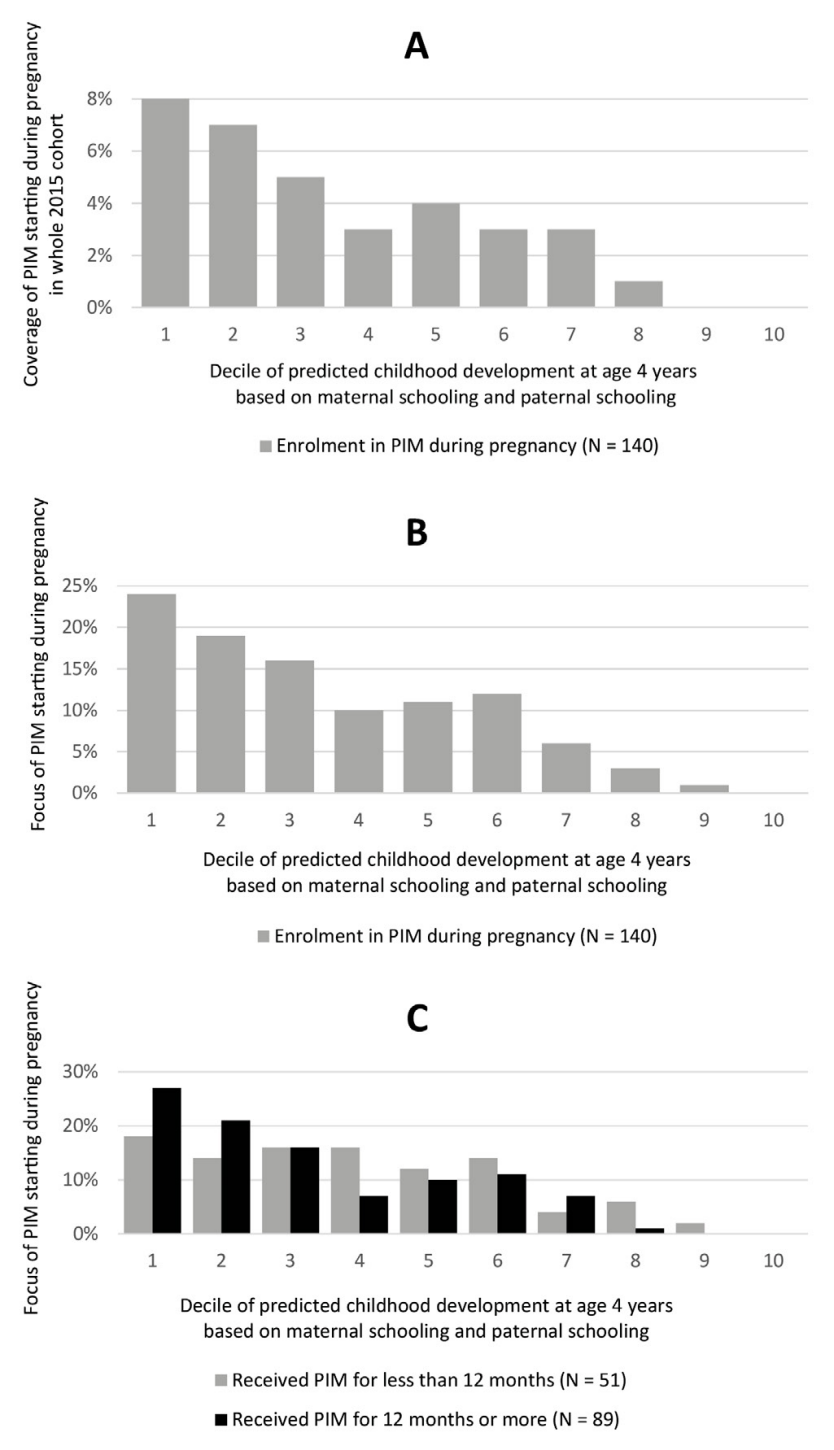
Coverage and focus of *PIM* starting during pregnancy across deciles of a predicted score of childhood development vulnerability at age 4 years based on maternal schooling and paternal schooling. **Panel A.** Coverage of *PIM* starting during pregnancy in the whole 2015 Pelotas Birth Cohort. **Panel B.** Focus of *PIM* starting during pregnancy. **Panel C.** Focus of *PIM* starting during pregnancy stratified according to duration of enrolment. *Coverage was measured as the proportion of children in each predicted BDI decile who did actually receive *PIM* starting in pregnancy. †Focus was measured as the proportion of children actually receiving *PIM* from pregnancy who belonged to each predicted BDI decile.

Of 716 children receiving *PIM* in the 2015 cohort analytical sample, 140 were enrolled before birth. To assess coverage and focus of the *PIM* starting during pregnancy, we stratified the sample into deciles of predicted BDI scores based on maternal and paternal schooling to obtain multiple groups with similar sizes. The *PIM* intervention starting during pregnancy presented higher coverage among more vulnerable deciles, but reached only 8% of children in the most vulnerable one (**Figure 5**, Panel A). Examining the focus of the programme starting during pregnancy, we saw that higher proportions of children receiving *PIM* were from more vulnerable deciles (**Figure 5**, Panel B). Importantly, there was a better focus when the duration of the intervention was longer (12 months or more) (**Figure 5**, Panel C).

### Post-hoc analysis including two perinatal predictors (gestational age and birth weight)

Adding gestational age and birth weight to the model as potential predictors did not change the decision tree results. Splits remained using maternal schooling first, followed by paternal schooling, and the same six final groups defined by these two variables were generated by the tree. Additionally, adjusted R^2^ statistics did not substantially improve in the linear regression (6.4%) or the PCA analyses (6.1%) when these two perinatal variables were added to the models. However, both perinatal predictors individually showed relevant positive associations with the outcome (Table S7–9 in the **Online Supplementary Document**), although these associations were weaker than those for parental schooling.

Although *PIM* and *PCF* aim to primarily enrol families during pregnancy, most are enrolled after birth. For this reason, we examined the coverage and focus of *PIM* for the subgroup of 576 children starting *PIM* after birth in the 2015 Pelotas cohort. We observed higher coverage (30%) in the most vulnerable decile of predicted BDI scores, based on maternal and paternal schooling, with the focus being similar to that for children starting PIM during pregnancy (Figure S8, Panels A–C in the **Online Supplementary Document**).

## DISCUSSION

We examined a wide range of potential predictors of child development that could be used to screen pregnant women to offer more vulnerable families support from a home visiting intervention. In this analysis, we did not identify a model with good discriminatory power in a large, population-based birth cohort study in Brazil. The strongest predictors in all three analytic approaches used were the number of completed years of maternal and paternal schooling. Including more predictors did not substantially improve a model's capacity to explain variance in child development at the age of four years. External validation of these results in a second, well-matched birth cohort showed consistent results. Considering child development vulnerability as indicated by levels of maternal and paternal schooling, coverage of *PIM* starting during pregnancy was low in more vulnerable deciles. However, the focus was adequate, especially because families receiving *PIM* for 12 months or more were more likely to belong to the most vulnerable group, indicating higher intervention dosage delivered to families most in need.

Although many predictors of child development have been identified across previous studies, maternal schooling is the most frequently retained variable in final predictive models [18,24,25,27,30], and often has the strongest predictive power [18,25,27]. Maternal education strongly predicted children’s low ECD scores (<10%) at four years of age in England (binary predictor; odds ratio (OR) = 2.56; 95% CI = 2.15, 3.04) [18] and their low IQ (SD equal to or less than −1) at age six years in Brazil (three category predictor; OR = 1.8; 95% CI = 1.6, 2.2) [25]. Only one study considered the use of paternal schooling in a predictive tool, in which the average of maternal and paternal years of schooling strongly predicted IQ among Danish children at the age of five years (predictor in years; β = 0.9; 95% CI = 0.5, 1.3) [27], indicating the need for more consideration of paternal characteristics regarding parental capital in the design and targeting of ECD interventions [51]. All prior studies used generalised linear models to select predictors, while we used decision tree methods and compared results with two traditional methods. The difference of around 0.8 SD in BDI scores between the decision tree extreme nodes (which were predicted by parental schooling in both 2015 and 2004 cohorts) has important implications for child life course outcomes in domains of education, work life, and physical and mental health into adulthood [1,2,52,53].

Prior longitudinal studies identified other strong predictors as measures of developmental milestones in periods before the final outcome [28–30]. Although early assessment of children’s developmental milestones is critical for postnatal targeting of interventions, this cannot be used to target interventions during pregnancy, which is when many programmes aim to first enrol participants. Maternal and paternal schooling, meanwhile, can be measured in pregnancy and are simple to assess at the population level, with low expected random error and recall bias. They are also both good markers of overall socioeconomic position, as well as of a home environment more responsive to the child and more keen on promoting early learning opportunities (i.e. complex cognitive experiences including language) [43,54–57].

Although only parental schooling consistently predicted child development scores in our study, we are not suggesting that determinants of child development are organised into a few or simple underlying mechanisms, nor that these mechanisms are not distinct enough to be understood singularly. They encompass many types of environmental exposures, as well as individual and age-related variations in sensitivity to the experiences of adversity. The complexity of predicting ECD outcomes requires holistic approaches considering many aspects of the environment. For example, children whose mothers are exposed to high psychosocial risk and socioeconomic disadvantage are particularly vulnerable to suboptimal development [58–60]. In line with this, our exploratory regression models highlighted those psychosocial risks, whereby maternal depression and maternal adverse childhood experiences were important predictors. Similarly, other risks may emerge during the postpartum period that warrant attention for future screening (e.g. gestational age, birth weight, and postpartum depression), with effective interventions to support parenting and child outcomes being available [1,8]. Nevertheless, before birth, risk factors representing socioeconomic deprivation may be more easily measurable than more complex psychosocial factors at the population level in LMICs.

Moreover, *PIM* did not enrol most of the more vulnerable pregnant women in this study population, indicating a missed opportunity for intervention during a sensitive period. This is particularly significant given that, in our previous evaluation of *PIM*, there was evidence of benefits for child development only when families were enrolled during pregnancy [32]. However, about half of the families enrolled in *PIM* during pregnancy and for the longest period (12 months or more) were from the two most vulnerable deciles of parental education, demonstrating a good focus of the programme when starting during pregnancy. It is noteworthy that, while there are benefits to enrolling mothers during pregnancy (e.g. building a trusting relationship with the home visitor, focussing on maternal concerns alongside readiness for parenting), the focus during pregnancy in other programmes and contexts with high risks in pregnancy may primarily be on maternal health care and safe delivery.

The main strengths of our study are the use of data from two prospective birth cohorts in the same city – representative of the entire population of the city and separated by an interval of 11 years – for initial testing and further external validation of the models; the wide range of potential predictors considered at the community and family levels, covering socioeconomic risks, psychosocial risks, and clinical health conditions during pregnancy; the fact that we did not miss any measurable predictors during pregnancy in a Brazilian PHC setting identified as strong in a previous study; and linking the 2015 cohort with *PIM* data to examine programme coverage and focus based on selected predictors, providing information that could be directly used to inform decision-making.

With these results in mind, we developed an online tool [61] to help managers prioritise pregnant women according to parental schooling to target in home visiting programmes in Brazil. The tool has two pages. On the first page (‘Pregnant women selection’), the user inputs the expected total number of new births in the population in one year and the proportion of those births that the programme intends to reach to obtain cutoffs of maternal and paternal schooling suggested by the tool. The second page (‘Technical details’) shows predicted scores of child development vulnerability according to all possible combinations of maternal and paternal years of schooling. For example, we can simulate the situation of aiming to improve focus of the *PCF* programme in Rio de Janeiro by inputting that there are 62 811 live births in the city per year (official number registered in 2023 [62]) and that the programme has resources to cover 5% of all pregnant women in the city. Using maternal-paternal education scores shown in the blue squares in the online tool (Figure S9 in the **Online Supplementary Document**) would then identify pregnant mothers where expected child development scores are −0.5 SD at age four years. Complementarily to the online tool, we provide the theoretical coverage and focus that would be achieved by the programme if targeting were based on a selected set of cutoff points (Box S3 in the **Online Supplementary Document**).

Our study also has some limitations. The BDI cutoff point used to define 10% of children with lower child development has not been validated. There is possible differential recall bias for the predictors examined (e.g. potential higher accuracy for maternal schooling than for maternal adverse childhood experiences), although we expect this would be similar in PHC real-life settings. Given missing data for the outcome variable, we did not include 14% of the original birth cohort in the main decision tree analysis that imputed missing predictor data only, leading to sample attrition. However, considering the baseline sample included almost the entire eligible population (98.7% response) and the good covariate balance between included and excluded participants, this relatively low attrition is unlikely to have caused significant bias. Decision trees are not the most suitable technique when interactions between covariates and nonlinear relationships are not present in the underlying structure of the data, as we observed here. There was an improvement in the population's parental schooling levels between 2004 and 2015 in Pelotas city [35], which made the cutoff point established in the 2015 cohort result in slightly greater sensitivity and lower specificity when applied to test external validation in the 2004 cohort sample. Further, while the representativeness of the sample is a critical issue, the educational level of the adult population in Pelotas city closely resembles that of Brazil as a whole [35]. Moreover, associations between selected predictors and ECD were consistent in the external validation in a sample born 11 years earlier, despite social and health transitions across the decade, while the applicability of the results across the country is facilitated by the uniform PHC structure, with similar team compositions, funding, goals, and information systems involved in prenatal health care across Brazil.

Examining additional predictors not tested in this study could potentially improve discriminatory power. For example, maternal IQ could represent an even stronger predictor than maternal schooling, while genetic factors might provide a deeper understanding of cognitive development across the life course [63]. However, both are not viable for population-level assessment in the Brazilian PHC context. Future investigations might develop and validate multifaceted screening tools for country-specific contexts to advance the targeting of ECD programmes.

## CONCLUSIONS

For an initial screening at the population level, the number of completed years of maternal and paternal schooling are useful for identifying more vulnerable pregnant women. This information could be collected relatively easily in PHC, which has frequent contact with vulnerable pregnant women and is widely accessed in Brazil. Pregnant women identified via such simple screening could then be referred for a first home visit to further consider the possibility of inclusion in the home visiting programme. However, eligibility should certainly not be solely determined by these two predictors (parental education) with low accuracy. They should not be the only criteria for inclusion, and when used should compose a comprehensive strategy that incorporates a holistic understanding of parental resources through in-person interactions with families in the home environment, allowing for joint decision on inclusion. More broadly, such strategies in the field of ECD must be implemented alongside support for education of adolescents and young adults in poorer communities, improving parental capital in a multi-generational strategy to break the cycle of poverty and improve human well-being.

## Additional material


Online Supplementary Document
