# Supplementary material for: Predictors of early child development for screening pregnant women most in need of support in Brazil
Source: J Glob Health. 2024 Aug 23;14:04143. doi: 10.7189/jogh.14.04143 (PMC11341113; doi:10.7189/jogh.14.04143)
Supplement: Online Supplementary Document [file jogh-14-04143-s001.pdf]

SUPPLEMENTARY MATERIAL

Child development measured at age 4 years

The screening version of Battelle's Developmental Inventory (BDI) was applied at the 4-year follow up in 2015 Pelotas Birth Cohort Study. This instrument consists of 96 items divided into five domains of neurodevelopment (personal-social, adaptive, motor, communication and cognitive) and can be used for children ranging in age from birth to 8 years [1]. This had previously been translated to Brazilian Portuguese and was adapted to form a reduced 66-item instrument (using all items for each age level from birth to 4–5 years of age, but excluding items for older ages) [2].

BDI was applied by trained interviewers who were supervised by senior psychologists. The instrument was divided into 13 questions for the mothers (applied first) and 53 items or fewer (depending on children's performance) that were directly applied to or observed with the children (without the mother's presence in the room). After applying the items assessing milestones for children aged 4–5 years and 3–4 years, which are unconditionally applied to all children, the evaluation continued with the application of items relevant to younger ages (first ages 2–3 years, then younger). The evaluation (of each domain) terminated when the child achieved the maximum score (2) for two consecutive items. At that point, items referring to lower-difficulty (younger age) skills were automatically scored as two points, as per the instrument instructions.

Quality control was performed in the 2015 cohort for 200 randomly selected children, through use of video recordings of the application of the instrument to the child, with re-coding being conducted by senior psychologists to calculate a total score. For all domains analysed, the agreement found was strong (kappa: 0.61 to 0.80) or excellent (kappa: 0.81 to 1) for age-appropriate questions, with the exception of 1 question of the motor domain (folds a paper twice; kappa: 0.49) and 1 question of the communication domain (follows verbal orders that imply two actions; kappa: 0.50). This pattern was maintained for questions referring to younger ages. The kappa coefficients refer to the agreement between the total score from coding by senior psychologists who observed the videos, taking into account the application environment, the interviewers' approach, and the children's responses, and the scores from the original interviewers' coding.

Conditional inference tree analysis

Decision trees are flexible statistical methods that can be used to explore combinations of predictors and non-linear relationships without having to test for all combinations. They first divide the sample into two smaller subgroups and continue to divide those two into even smaller subgroups until a stopping rule is triggered. The conditional inference tree (CTree) executes the partition process in two steps using formal hypothesis tests. First, it chooses the predictor most strongly associated with the outcome in the sample (the covariate to be used to split the sample) based on the strength of bivariate association with the outcome, which is defined by the minimum asymptotic p-value for the conditional distribution of test statistic. The selection of the covariate in that first step avoids a variable selection bias towards variables with many possible cutoff points or with missing data. Subsequently, in the second step of the partition process, the split point (cutoff point) for the selected covariate is defined by a maximised test statistic that is selected according to the predictor and outcome's variable types, in order to maximise the difference between the two subgroups created by the split [3–5].

For this study, the stop criterion in step 1 was based on multiplicity adjusted p-values (testtype = "Bonferroni"). When the minimum Bonferroni-adjusted p-value available was larger than the significance threshold (< 0.05), the node was declared a terminal group, thus reducing the overfitting problem. This statistical approach aims to ensure that the right-sized tree is grown without additional pruning criteria [3,5].

The analytical sample of this study included all individuals having data for the outcome BDI at the 4-year follow-up of the 2015 Pelotas Birth Cohort Study. In case of missing data for any potential predictor, CTree applied surrogate splits. We specify the parameter "maxsurrogate = 5", allowing the algorithm to find surrogates for all individuals with missing data for potential predictors. In addition, the minimum size of any group was set to be 50 children. All other standard parameters of *partykit* package in R version 4.1.0 were kept.

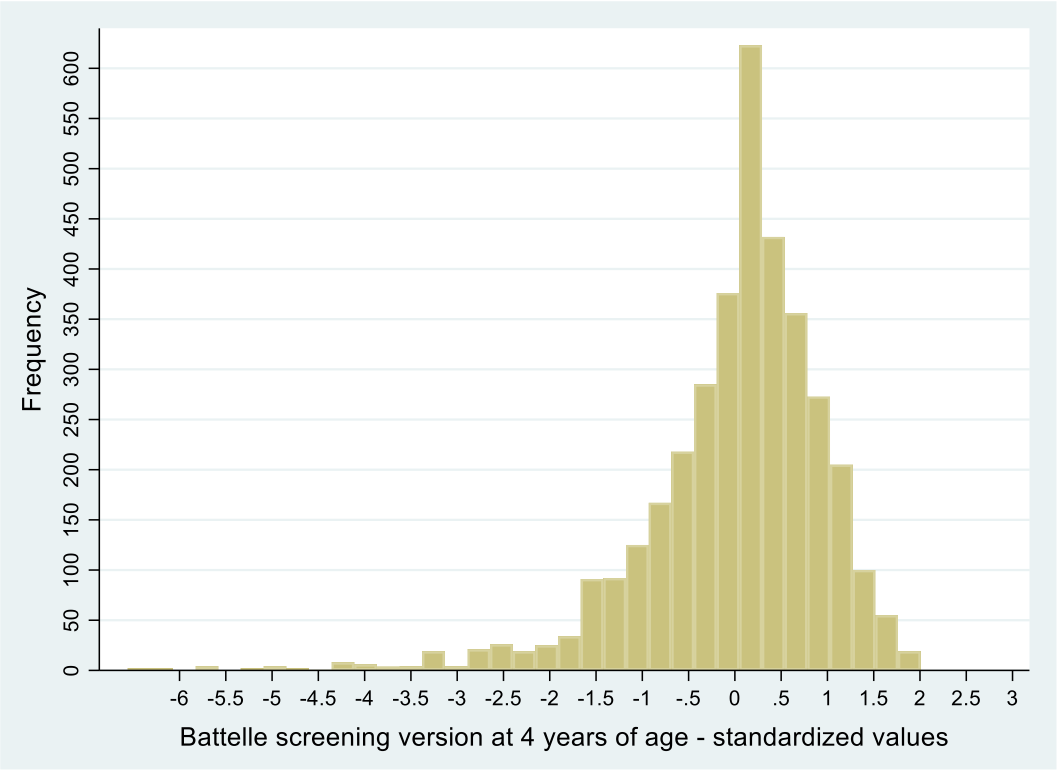

Figure S1. Distribution of standardised Battelle Developmental Inventory (screening version) in 2015 Pelotas Birth Cohort (N = 3603).

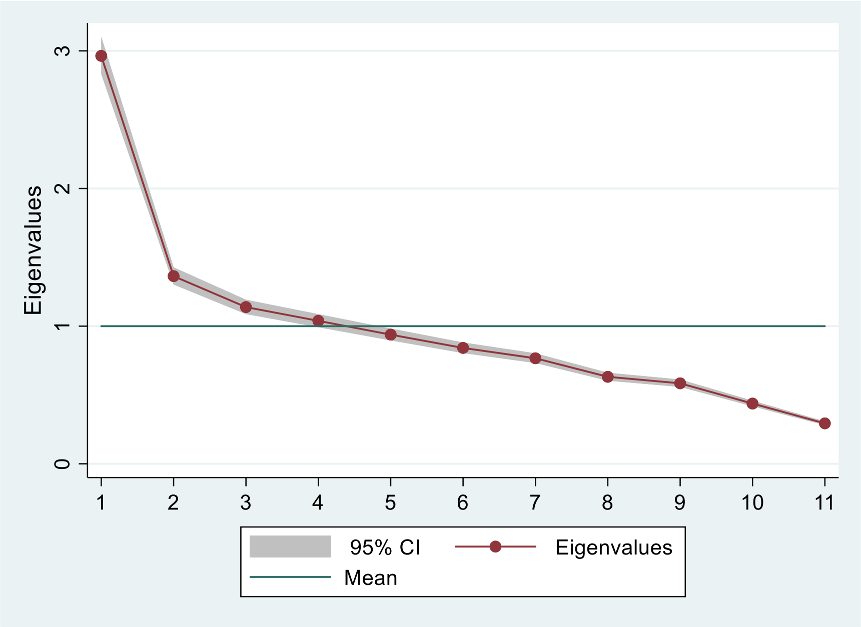

Figure S2. Scree plot of eigenvalues after Principal Components Analysis considering 11 moderately to highly correlated potential predictors in 2015 Pelotas Birth Cohort.

A

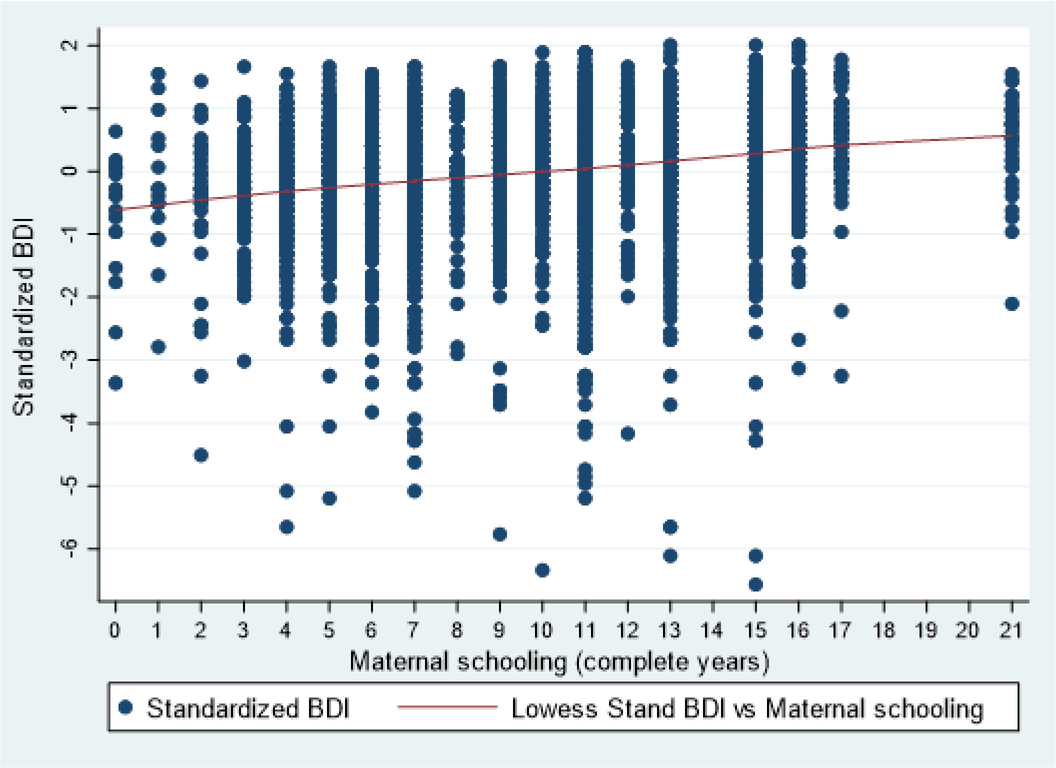

Standardized BDI
2
1
0
-1
-2
-3
-4
-5
-6
0 1 2 3 4 5 6 7 8 9 10 11 12 13 14 15 16 17 18 19 20 21
Maternal schooling (complete years)
Standardized BDI
Lowess Stand BDI vs Maternal schooling

B

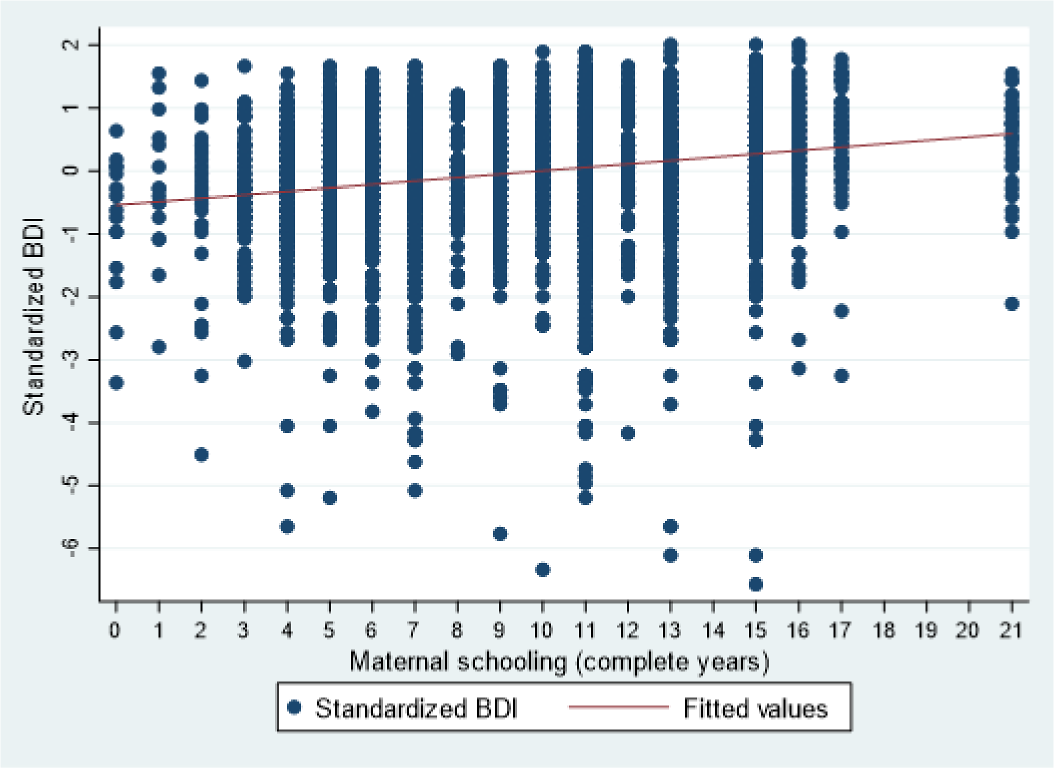

Standardized BDI
2
1
0
-1
-2
-3
-4
-5
-6
0 1 2 3 4 5 6 7 8 9 10 11 12 13 14 15 16 17 18 19 20 21
Maternal schooling (complete years)
Standardized BDI
Fitted values

C

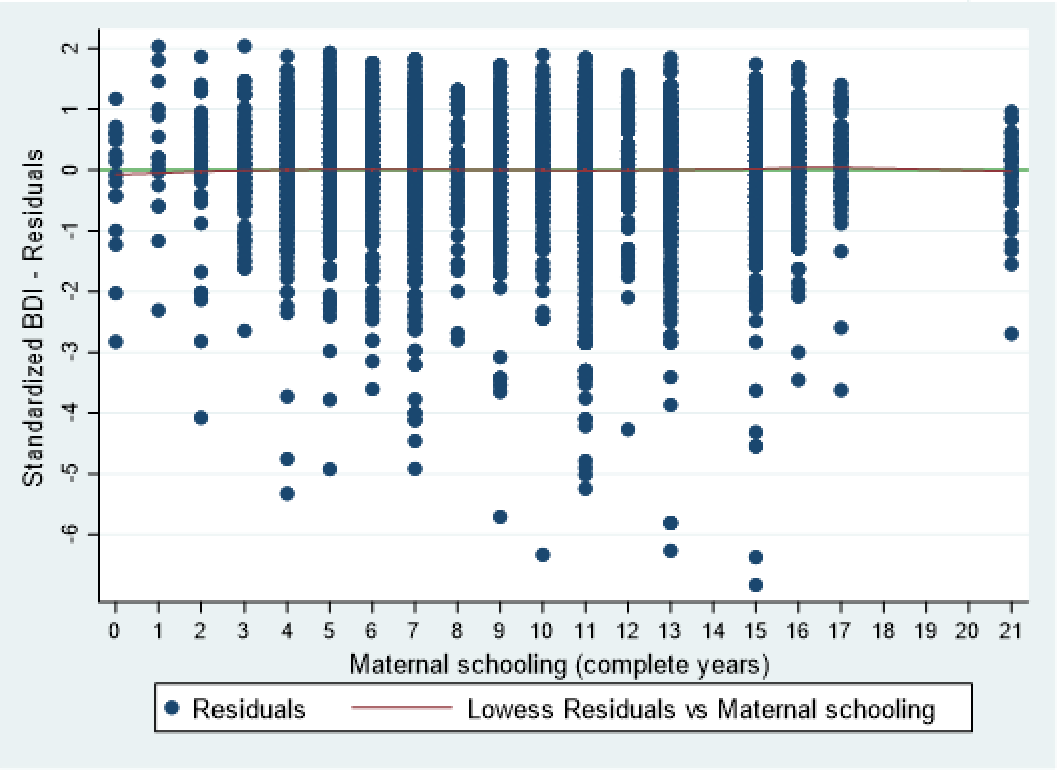

Figure S3. Examining linear association between Maternal schooling and Standardised Battelle Developmental Inventory (screening version) at age 4 years in the 2015 Pelotas Birth Cohort. A (red line represents smoothing line); B (red line represents linear fitted values); C (red line represents smoothing line against residuals).

A

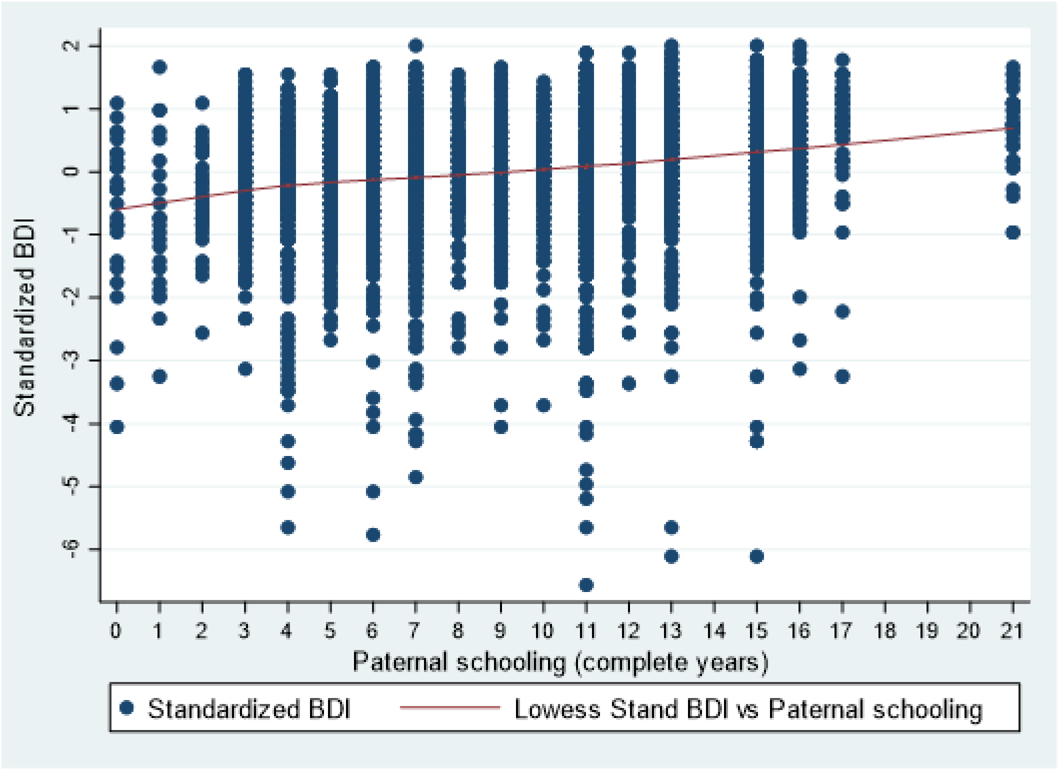

B

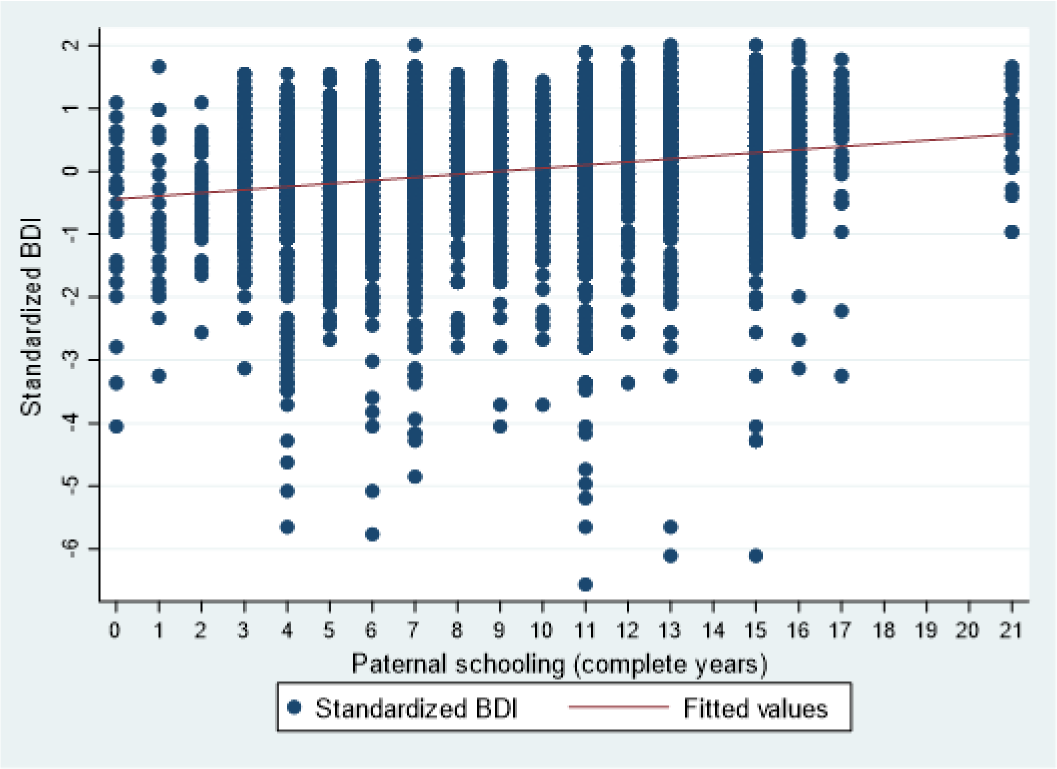

C

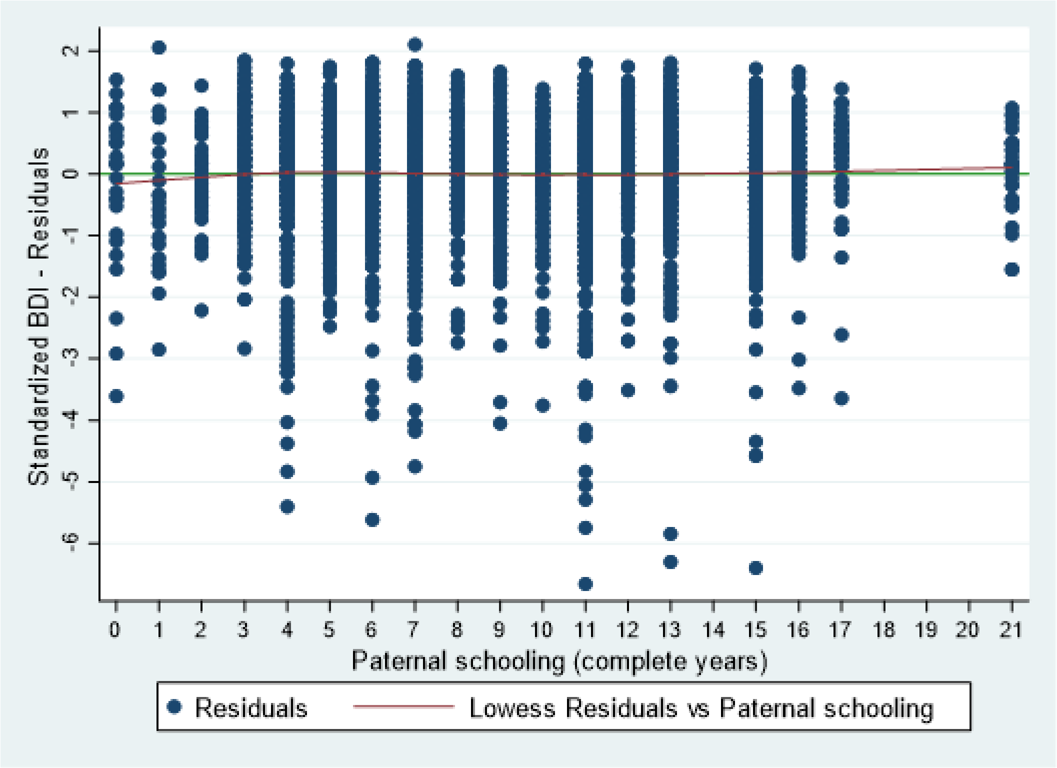

Figure S4. Examining linear association between Paternal schooling and Standardised Battelle Developmental Inventory (screening version) at age 4 years in the 2015 Pelotas Birth Cohort. A (red line represents smoothing line); B (red line represents linear fitted values); C (red line represents smoothing line against residuals).

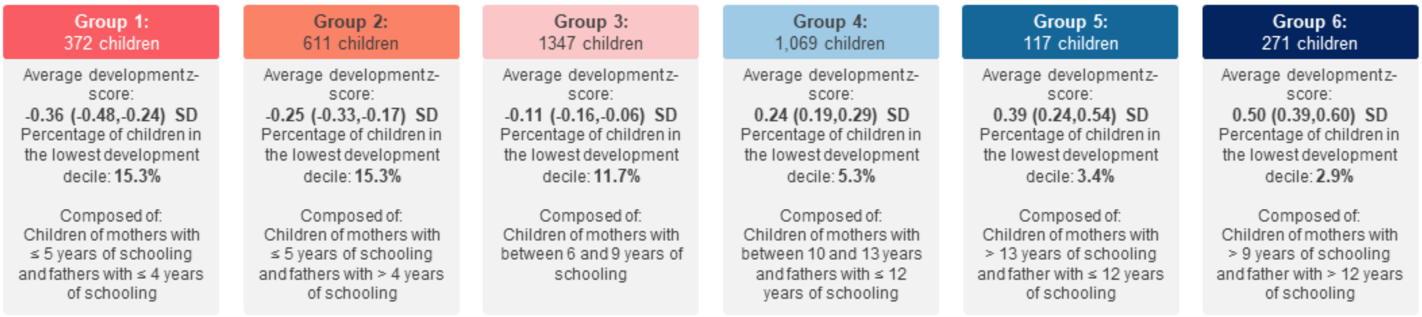

Supplemental Figure S5. External validation of the partition rules (generated with 2015 Pelotas Birth Cohort data) in the 2004 Pelotas Birth Cohort data. Outcome distribution within decision tree terminal groups (N = 3787).

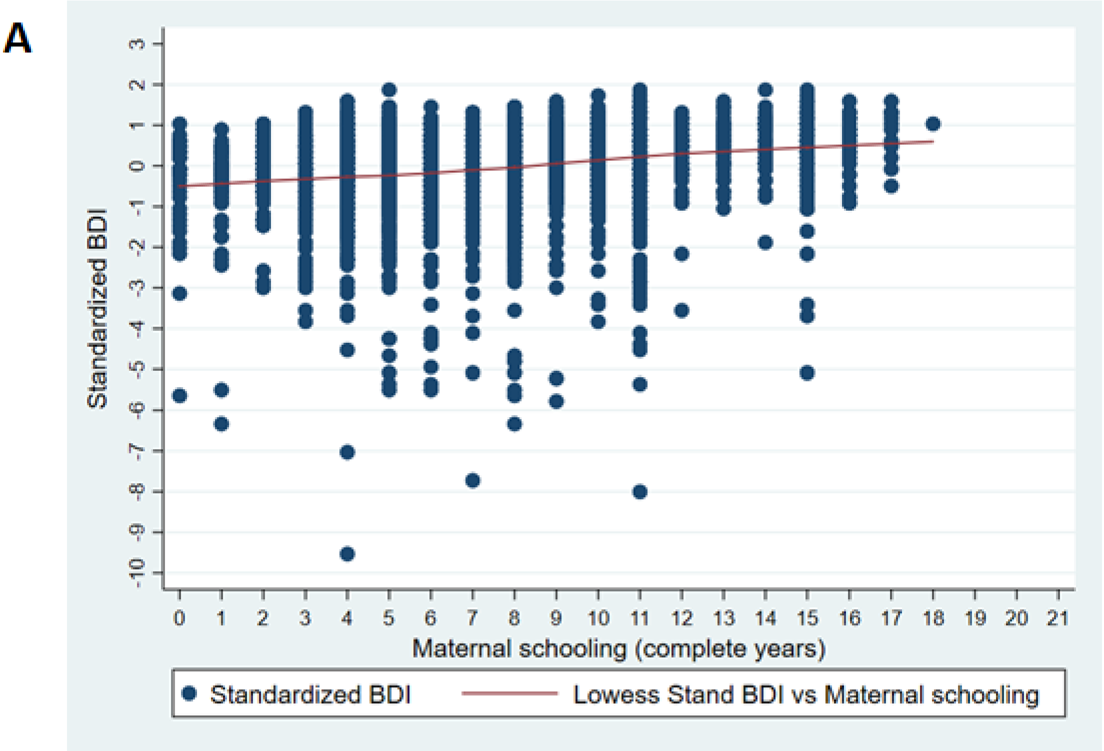

B

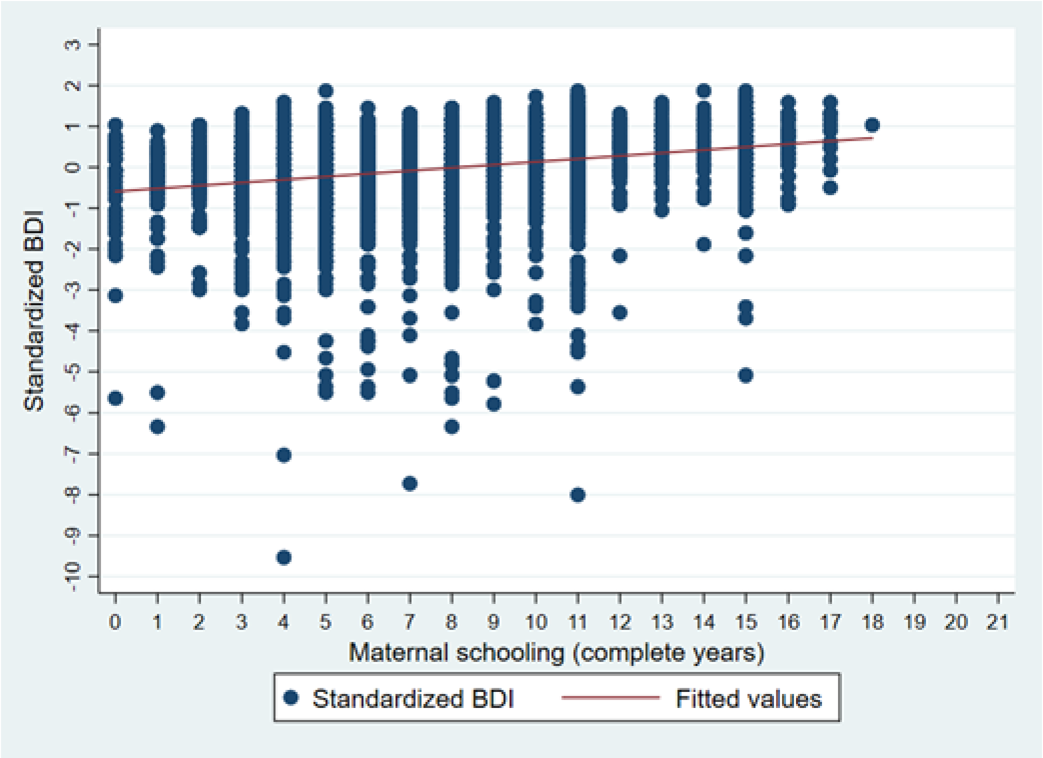

C

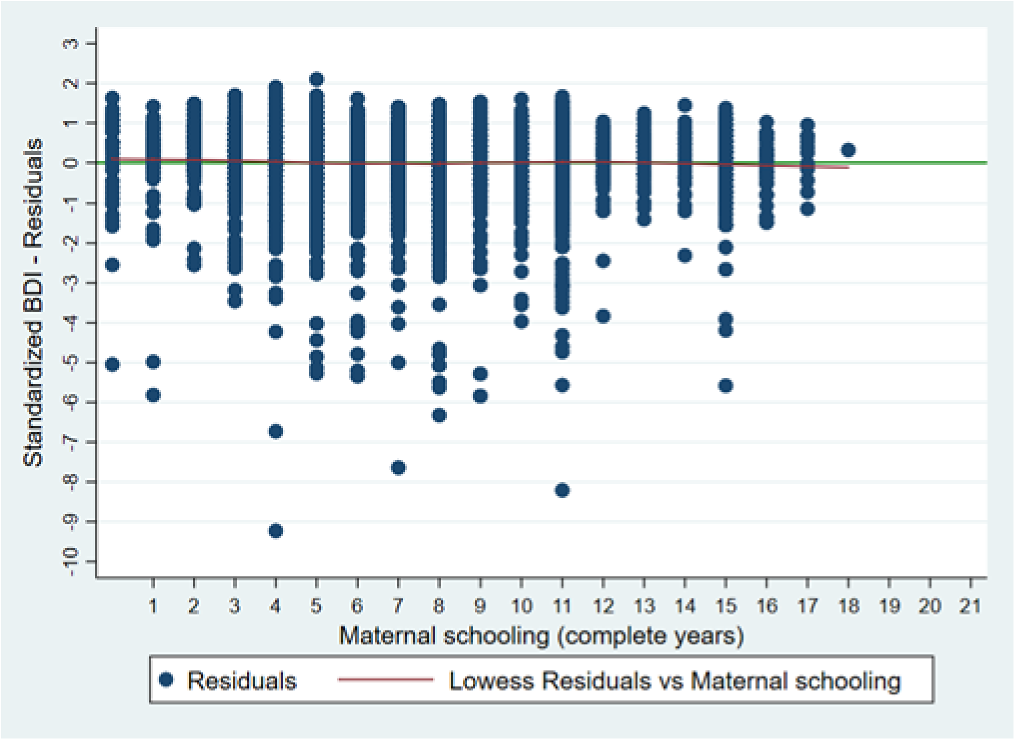

Figure S6. Examining linear association between Maternal schooling and Standardised Battelle Developmental Inventory (screening version) at age 4 years in the 2004 Pelotas Birth Cohort. A (red line represents smoothing line); B (red line represents linear fitted values); C (red line represents smoothing line against residuals).

A

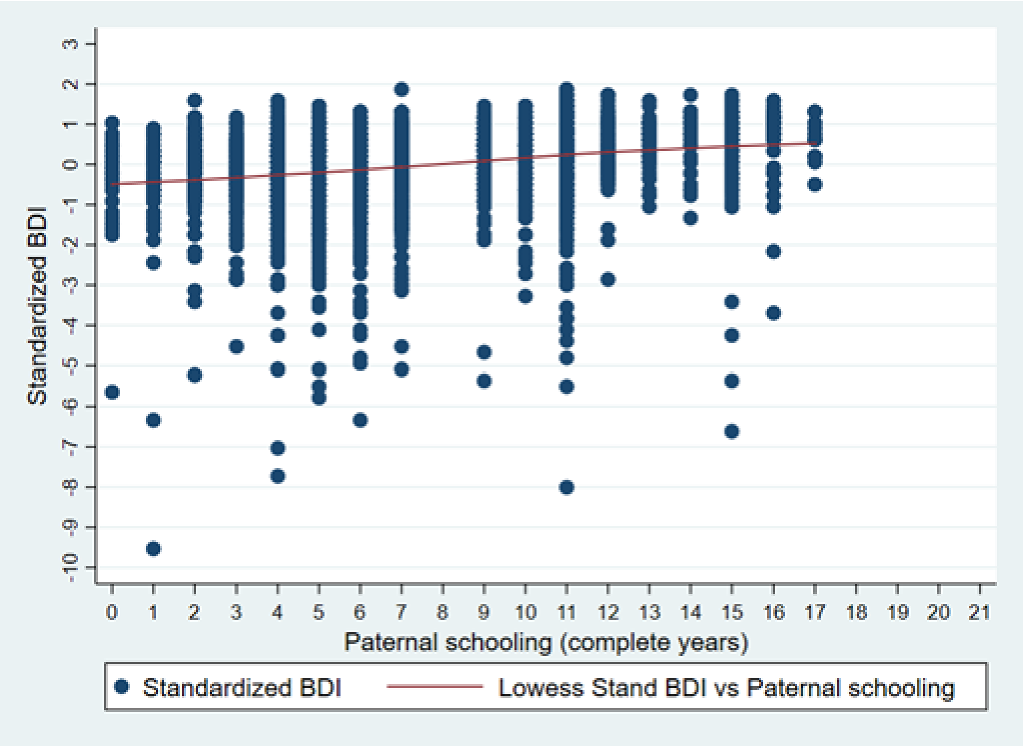

Standardized BDI
Paternal schooling (complete years)
Standardized BDI
Lowess Stand BDI vs Paternal schooling

B

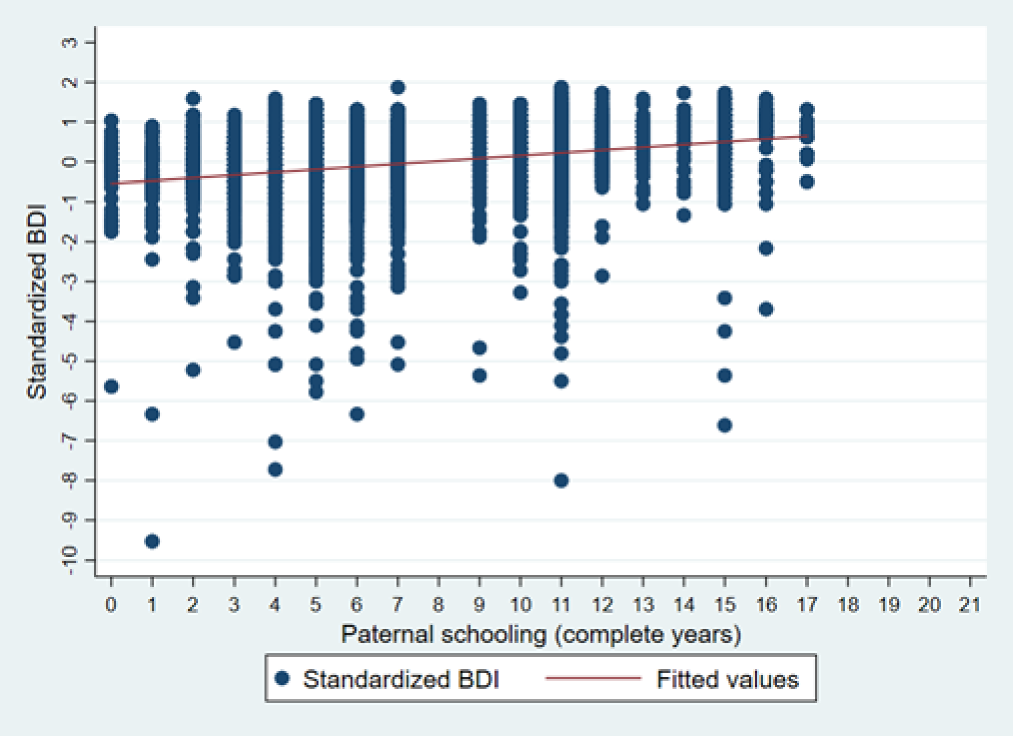

Standardized BDI
Paternal schooling (complete years)
Standardized BDI
Fitted values

C

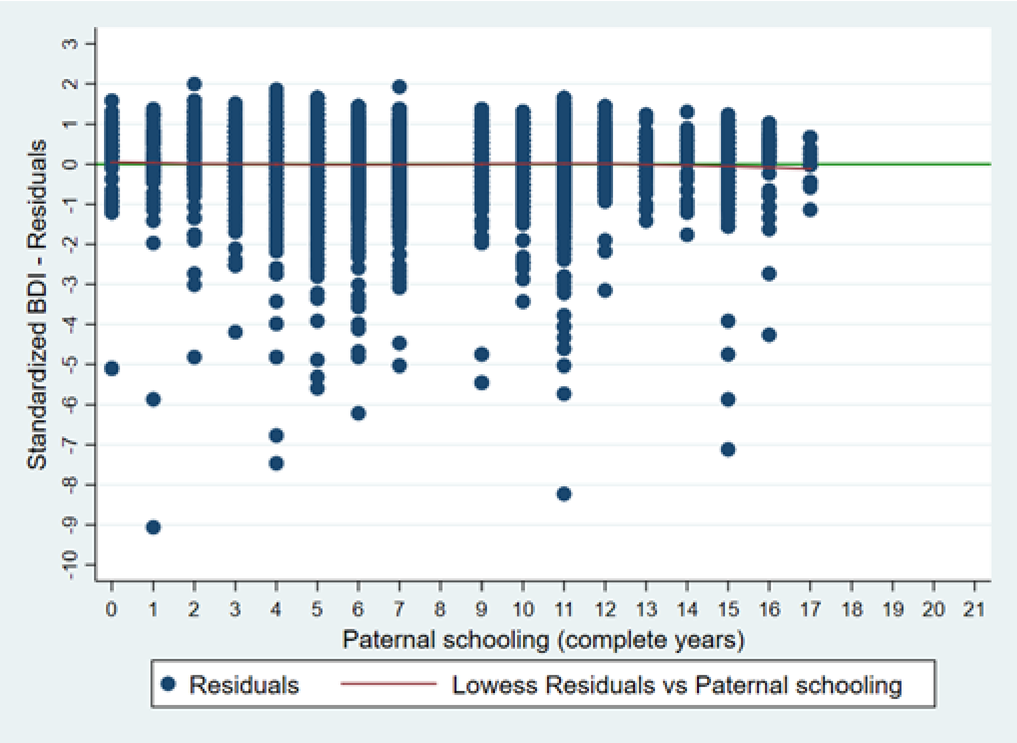

Figure S7. Examining linear association between Paternal schooling and Standardised Battelle Developmental Inventory (screening version) at age 4 years in the 2004 Pelotas Birth Cohort. A (red line represents smoothing line); B (red line represents linear fitted values); C (red line represents smoothing line against residuals).

A

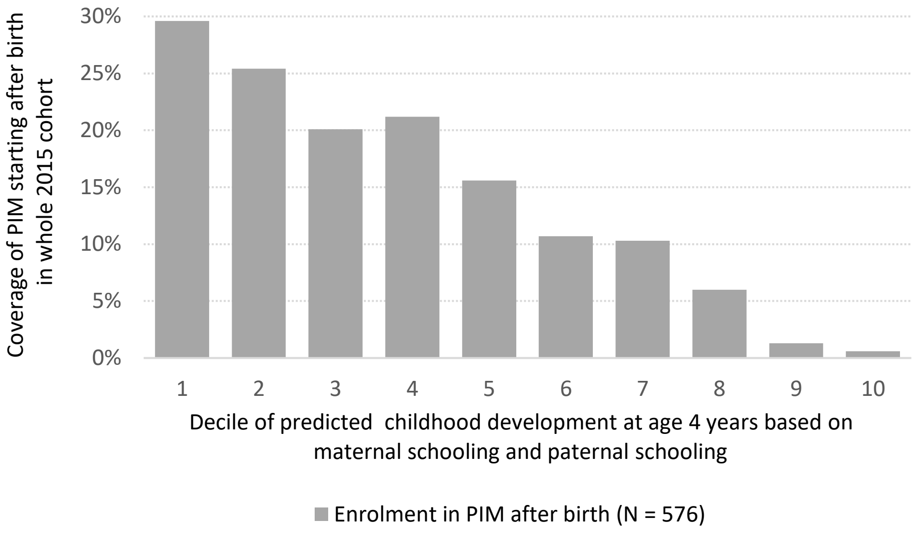
Coverage of PIM starting after birth in whole 2015 cohort
30%
25%
20%
15%
10%
5%
0%
1
2
3
4
5
6
7
8
9
10
Decile of predicted childhood development at age 4 years based on maternal schooling and paternal schooling
Enrolment in PIM after birth (N = 576)

B

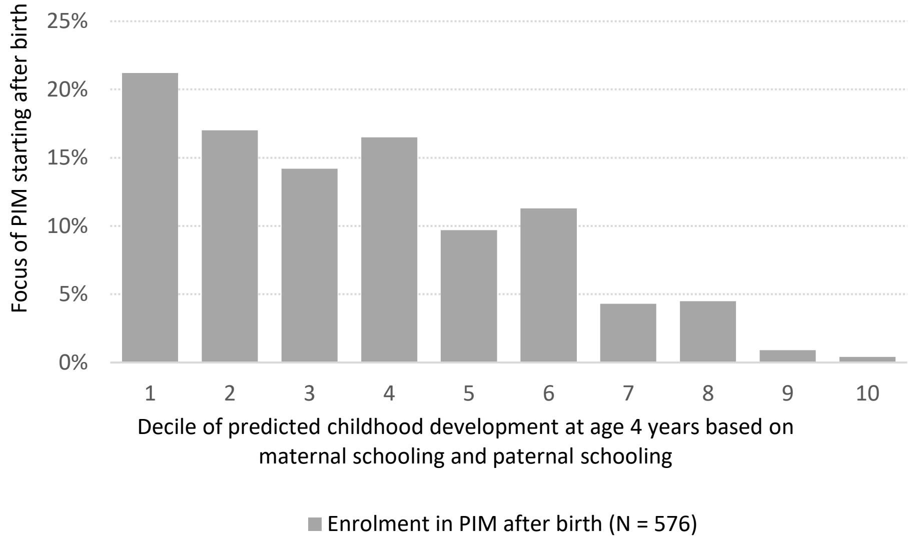
Focus of PIM starting after birth
25%
20%
15%
10%
5%
0%
1
2
3
4
5
6
7
8
9
10
Decile of predicted childhood development at age 4 years based on maternal schooling and paternal schooling
Enrolment in PIM after birth (N = 576)

C

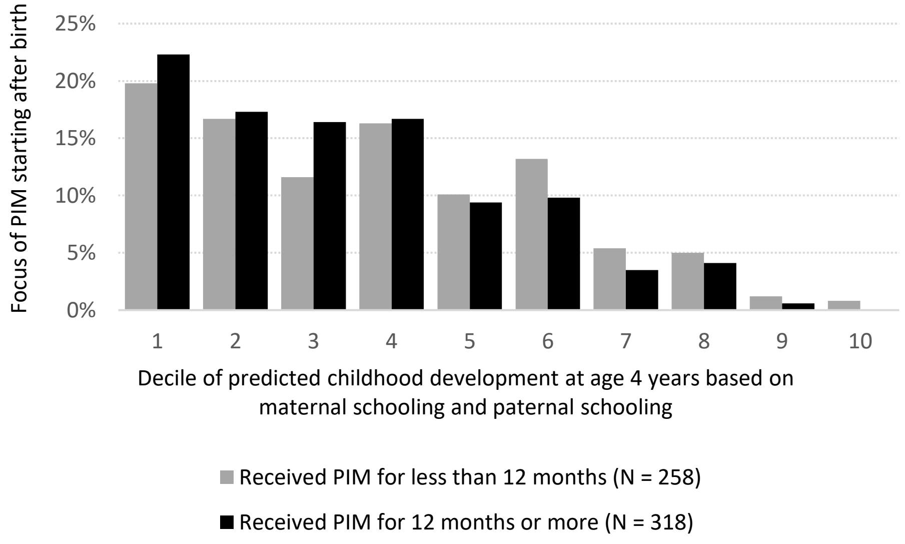
Focus of PIM starting after birth
25%
20%
15%
10%
5%
0%
1
2
3
4
5
6
7
8
9
10
Decile of predicted childhood development at age 4 years based on maternal schooling and paternal schooling
Received PIM for less than 12 months (N = 258)
Received PIM for 12 months or more (N = 318)

Figure S8. Coverage and focus of Primeira Infância Melhor (PIM) starting after birth across deciles of a predicted score of childhood development vulnerability at age 4 years based on maternal schooling and paternal schooling.
Panel A - Coverage of PIM starting after birth in the whole 2015 Pelotas Birth Cohort.
Panel B - Focus of PIM starting after birth.
Panel C - Focus of PIM starting after birth stratified according to duration of enrolment.
*****Coverage was measured as the proportion of children in each predicted BDI decile who did actually receive PIM starting after birth.
******Focus was measured as the proportion of children actually receiving PIM starting after birth who belonged to each predicted BDI decile.

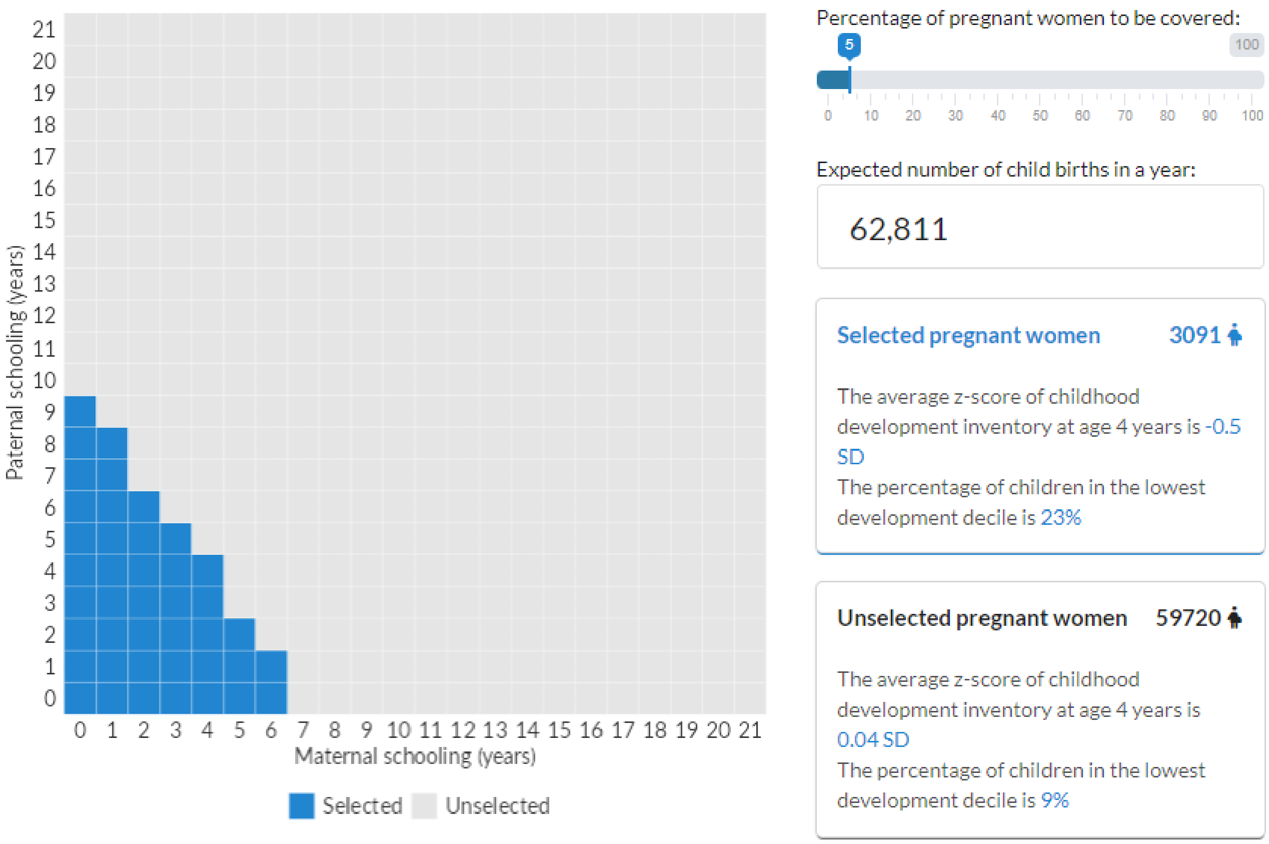

Figure S9. Applied example using the *Initial Screening Tool: Selecting families for early childhood development programmes.*
The image shows a simulation aimed at enhancing the focus of the Programa Criança Feliz (PCF programme) in Rio de Janeiro. Two informations were inputted into the tool: there are 62,811 live births in the city per year (official number registered in 2023), and the programme has resources to cover 5% of all pregnant women in the city. Using all combinations of maternal-paternal education scores represented in the blue squares would then identify 3,091 pregnant mothers where expected child development scores are -0.5 SD at age 4 years.
The online tool is available at https://ecdprograms.shinyapps.io/ECDPrograms/.

Table S1. Comparison of characteristics between total 2015 Pelotas Birth Cohort sample, and analytical sample for conditional inference tree analysis.

	Whole cohort (N = 4275)	Analytic Sample (N = 3603)
	**n(%)**	**n(%)**
**Maternal skin colour**		
White	3024(70.9)	2530(70.3)
Black	667(15.6)	583(16.2)
Brown	551(12.9)	462(12.8)
Asian	16(0.4)	13(0.4)
Indigenous	10(0.2)	9(0.3)
Missing data (n)	7	6
**Paternal skin colour**		
White	2983(70.9)	2498(70.5)
Black	653(15.5)	571(16.1)
Brown	541(12.9)	455(12.8)
Asian	11(0.3)	7(0.2)
Indigenous	17(0.4)	15(0.4)
Missing data (n)	70	57
**Neighbourhood violence**		
Low	2320(58.3)	2061(57.5)
Medium	1343(33.7)	1227(34.2)
High	320(8.0)	296(8.3)
Missing data (n)	292	19
**Garbage accumulated in the neighborhood**		
No	1614(51.6)	1392(51.5)
Yes	1514(48.4)	1309(48.5)
Missing data (n)	1147	902
**Open sewer in the neighborhood**		
No	1614(51.6)	1391(51.5)
Yes	1512(48.4)	1308(48.5)
Missing data (n)	1149	904
**Maternal drug use during pregnancy**		
No	3100(98.9)	2678(98.9)
Yes	35(1.1)	29(1.1)
Missing data (n)	1140	896
**Maternal alcohol use during pregnancy**		
No	3957(92.6)	3335(92.6)
Yes	315(7.4)	265(7.4)
Missing data (n)	3	3
**Mother smoked during pregnancy**		
No	3640(85.2)	3077(85.5)
Yes	631(14.8)	522(14.5)
Missing data (n)	4	4
**Mother's partner smokes at perinatal**		
No	2798(76.3)	2377(77.0)

Yes	869(23.7)	712(23.0)
Missing data (n)	608	514
**Prenatal maternal depressive symptoms**		
0 a 10	2377(75.9)	2051(75.9)
11 or more	755(24.1)	653(24.1)
Missing data (n)	1143	899
**Postnatal maternal depressive symptoms**		
0 a 9	3264(79.7)	2800(79.1)
10 or more	831(20.3)	740(20.9)
Missing data (n)	180	63
**People per bedroom in the house**		
≤ 2	1678(40.9)	1430(40.3)
> 2 ≤ 3	1757(42.8)	1541(43.5)
> 3 ≤ 4	482(11.8)	410(11.6)
> 4	186(4.5)	165(4.7)
Missing data (n)	172	57
**Number of children living with the mother**		
0	2174(50.9)	1821(50.7)
1	1341(31.4)	1136(31.5)
2	453(10.6)	383(10.6)
3	169(4.0)	143(4.0)
4 or more	137(3.2)	113(3.1)
Missing data (n)	1	1
**Planned pregnancy**		
Yes	2058(48.2)	1748(48.5)
No	2216(51.9)	1854(51.5)
Missing data (n)	1	1
**Father's support level during pregnancy**		
Little or none support	224(5.3)	200(5.6)
Medium support	238(5.7)	198(5.6)
Much support	3736(89.0)	3146(88.8)
Missing data (n)	77	59
**Couple relationship characterized by criticism**		
Low criticism	2231(64.8)	1936(65.1)
Medium criticism	711(20.7)	612(20.6)
High criticism	501(14.6)	426(14.3)
Missing data (n)	832	629
**Mother living with partner at birth**		
No	607(14.2)	513(14.2)
Yes	3667(85.8)	3089(85.8)
Missing data (n)	1	1
**Mother worked during pregnancy**		
No	1895(44.3)	1561(43.3)
Yes	2379(55.7)	2041(56.7)
Missing data (n)	1	1
**Father working at perinatal**		
No	540(12.9)	454(12.9)
Yes	3634(87.1)	3065(87.1)
Missing data (n)	101	84

**Maternal diabetes during pregnancy**		
No	3906(91.4)	3286(91.3)
Yes	366(8.6)	314(8.7)
Missing data (n)	3	3
**Maternal hipertension during pregnancy**		
No	3183(74.5)	2685(74.6)
Yes	1089(25.5)	915(25.4)
Missing data (n)	3	3
**Maternal anemia during pregnancy**		
No	3717(87.4)	3121(87.2)
Yes	534(12.6)	459(12.8)
Missing data (n)	24	23
	**Mean (SD)**	
**Maternal age at birth (years)**	27.6(6.6)	27.7(6.6)
Missing data (n)	1	0
**Maternal schooling (years)**	10.0(4.0)	10.0(4.0)
Missing data (n)	1	1
**Paternal schooling (years)**	9.4(4.1)	9.3(4.0)
Missing data (n)	252	209
**Family income (minimum wage)**	3.9(5.3)	3.8(5.6)
Missing data (n)	2	2
**Maternal Adverse Childhood Experiences**	1.8(1.7)	1.8(1.7)
Missing data (n)	303	26

Table S2. Unadjusted association between potential predictors and early childhood development at age 4 years in 2015 Pelotas Birth Cohort.

	Standardized BDI - total score	
	**β***	**95% CI**
**Maternal skin colour (n = 3597)**		**p < 0.001**
White	0	-
Black	-0.23	-0.32 to -0.14
Brown	-0.21	-0.31 to -0.11
Asian	-0.24	-0.78 to 0.30
Indigenous	-0.71	-1.37 to -0.06
**Paternal skin colour (n = 3546)**		**p < 0.001**
White	0	-
Black	-0.13	-0.22 to -0.04
Brown	-0.26	-0.36 to -0.16
Asian	0.49	-0.24 to 1.23
Indigenous	-0.71	-1.22 to -0.21
**Neighbourhood violence (n = 3584)**		**p = 0.037**
Low	0	-
Medium	-0.09	-0.16 to -0.02
High	-0.06	-0.18 to 0.06
**Garbage accumulated in the neighborhood (n = 2701)**		**p = 0.407**
No	0	-
Yes	-0.03	-0.11 to 0.04
**Open sewer in the neighborhood (n = 2699)**		**p = 0.199**
No	0	-
Yes	-0.05	-0.12 to 0.03
**Maternal drug use during pregnancy (n = 2707)**		**p = 0.859**
No	0	-
Yes	-0.03	-0.39 to 0.33
**Maternal alcohol use during pregnancy (n = 3600)**		**p = 0.172**
No	0	-
Yes	-0.09	-0.21 to 0.04
**Mother smoked during pregnancy (n = 3599)**		**p < 0.001**
No	0	-
Yes	-0.19	-0.28 to -0.10
**Mother's partner smokes at perinatal (n = 3089)**		**p < 0.001**
No	0	-
Yes	-0.21	-0.30 to -0.13
**Prenatal maternal depressive symptoms (n = 2704)**		**p < 0.001**
0 a 10	0	-
11 or more	-0.19	-0.28 to -0.11
**Postnatal maternal depressive symptoms (n = 3540)**		**p = 0.001**
0 a 9	0	-
10 or more	-0.13	-0.21 to -0.05
**People per bedroom in the house (n = 3546)**		**p = 0.001**

≤ 2	0	-
> 2 ≤ 3	-0.03	-0.11 to 0.04
> 3 ≤ 4	-0.19	-0.29 to -0.08
> 4	-0.25	-0.41 to -0.09
**Number of children living with the mother (n = 3602)**		**p = 0.021**
0	0	-
1	-0.03	-0.10 to 0.05
2	-0.12	-0.23 to -0.01
3	-0.17	-0.34 to -0.00
4 or more	-0.21	-0.40 to -0.02
**Planned pregnancy (n = 3602)**		**p = 0.001**
Yes	0	-
No	-0.12	-0.18 to -0.05
**Father's support level during pregnancy (n = 3544)**		**p = 0.005**
Little or none support	0	-
Medium support	0.05	-0.15 to 0.24
Much support	0.20	0.05 to 0.34
**Couple relationship characterized by criticism (n = 2974)**		**p = 0.092**
Low criticism	0	-
Medium criticism	-0.10	-0.19 to -0.01
High criticism	-0.06	-0.16 to 0.05
**Mother living with partner at birth (n = 3602)**		**p < 0.001**
No	0	-
Yes	0.18	0.09 to 0.27
**Mother worked during pregnancy (n = 3602)**		**p < 0.001**
No	0	-
Yes	0.24	0.18 to 0.31
**Father working at perinatal (n = 3519)**		**p = 0.022**
No	0	-
Yes	0.12	0.02 to 0.21
**Maternal diabetes during pregnancy (n = 3600)**		**p = 0.084**
No	0	-
Yes	-0.10	-0.22 to 0.01
**Maternal hipertension during pregnancy (n = 3600)**		**p = 0.029**
No	0	-
Yes	-0.08	-0.16 to -0.01
**Maternal anemia during pregnancy (n = 3580)**		**p = 0.556**
No	0	-
Yes	-0.03	-0.13 to 0.07
**Maternal age at birth (years) (n = 3603)**	0.01	**p < 0.001** 0.00 to 0.01
**Maternal schooling (years) (n = 3602)**	0.05	**p < 0.001** 0.05 to 0.06
**Paternal schooling (years) (n = 3394)**	0.05	**p < 0.001** 0.04 to 0.06
**Family income (minimum wage) (n = 3601)**		**p < 0.001**

	0.02	0.01 to 0.03
**Maternal Adverse Childhood Experiences (n = 3577)**		**p = 0.001**
	-0.03	-0.05 to -0.01

*Unadjusted linear regression

Table S3. Proportion of variance explained by each of the four Principal Components and eigenvectors for 11 predictors within each Principal Component.

**Predictor**	**Comp1**<br>**27% of variance**	**Comp2**<br>**13% of variance**	**Comp3**<br>**10% of variance**	**Comp4**<br>**9% of variance**
Maternal age (<20/20+)	0.18	**0.59**	0.27	-0.28
Maternal schooling (years)	**0.49**	0.06	0.20	0.11
Paternal schooling (years)	**0.46**	0.06	0.16	0.16
Family income (minimum wage quintile)	**0.43**	0.11	0.16	0.11
Maternal skin colour (white/non-white)	-0.27	-0.17	**0.58**	-0.08
Paternal skin colour (white/non-white)	-0.24	-0.20	**0.63**	-0.10
Number of children living with the mother (1/2/3/4+)	-0.21	**0.58**	0.07	**-0.33**
Mother smoked during pregnancy (no/yes)	-0.24	**0.33**	-0.10	0.28
Maternal alcohol use during pregnancy (no/yes)	-0.09	0.14	0.28	**0.74**
Maternal Adverse Childhood Experiences (frequency – 1 to 9)	-0.22	0.20	-0.09	0.29
Postnatal maternal depressive symptoms (low/moderate/significant)	-0.21	0.24	-0.04	0.21

Table S4. Results of fractional polynomial analysis examining the linearity of the association between maternal schooling (complete years) and standardised BDI, and between paternal schooling (complete years) and standardised BDI, in the 2015 Pelotas Birth Cohort. Deviances for 44 combinations of two exponential terms (out of a series of eight: -2; -1; -0.5; 0; 0.5; 1; 2; 3) used by the standard Stata's package are presented.

Maternal schooling*

Order in Stata's output	Deviance	Difference from the best model	Exponent	Exponent
14	10056.97	0.00	1.0	-2.0
21	10057.22	0.26	1.0	-1.0
33	10057.31	0.34	2.0	0.0
27	10057.42	0.46	1.0	-0.5
37	10057.50	0.53	2.0	0.5
38	10057.60	0.63	3.0	0.5
32	10057.62	0.65	1.0	0.0
28	10057.74	0.78	2.0	-0.5
36	10057.77	0.80	1.0	0.5
39	10057.86	0.90	1.0	1.0
40	10057.94	0.98	2.0	1.0
41	10057.96	0.99	3.0	1.0
**6**	**10058.00**	**1,03**	**1.0**	**.**
35	10058.07	1.11	0.5	0.5
34	10058.25	1.29	3.0	0.0
31	10058.46	1.49	0.5	0.0
42	10058.82	1.85	2.0	2.0
26	10058.84	1.88	0.5	-0.5
22	10058.90	1.93	2.0	-1.0
20	10059.13	2.17	0.5	-1.0
13	10059.36	2.39	0.5	-2.0
5	10059.37	2.41	0.5	.
43	10059.46	2.50	3.0	2.0
30	10059.77	2.80	0.0	0.0
29	10061.00	4.04	3.0	-0.5
44	10061.09	4.13	3.0	3.0
25	10061.37	4.41	0.0	-0.5

Paternal schooling**

Order in Stata's output	Deviance	Difference from the best model	Exponent	Exponent
22	9432.23	0.00	2.0	-1.0
28	9432.26	0.03	2.0	-0.5
34	9432.52	0.29	3.0	0.0
29	9432.53	0.30	3.0	-0.5
33	9433.38	1.15	2.0	0.0
15	9434.10	1.87	2.0	-2.0
38	9434.21	1.98	3.0	0.5
14	9434.50	2.27	1.0	-2.0
21	9434.77	2.54	1.0	-1.0
37	9435.00	2.77	2.0	0.5
23	9435.03	2.80	3.0	-1.0
27	9435.29	3.06	1.0	-0.5
32	9435.92	3.69	1.0	0.0
41	9436.21	3.98	3.0	1.0
36	9436.40	4.17	1.0	0.5
40	9436.46	4.23	2.0	1.0
39	9436.59	4.36	1.0	1.0
**6**	**9436.61**	**4.38**	**1.0**	**.**
35	9437.12	4.89	0.5	0.5
31	9437.64	5.41	0.5	0.0
13	9437.83	5.60	0.5	-2.0
26	9437.89	5.66	0.5	-0.5
20	9437.92	5.69	0.5	-1.0
5	9437.92	5.69	0.5	.
42	9438.32	6.09	2.0	2.0
43	9439.21	6.98	3.0	2.0
30	9439.37	7.14	0.0	0.0

15	10061.41	4.45	2.0	-2.0	25	9440.83	8.60	0.0	-0.5
19	10062.97	6.00	0.0	-1.0	44	9441.07	8.85	3.0	3.0
24	10064.73	7.77	-0.5	-0.5	16	9441.77	9.54	3.0	-2.0
12	10065.27	8.30	0.0	-2.0	7	9441.87	9.64	2.0	.
23	10065.98	9.01	3.0	-1.0	19	9441.89	9.67	0.0	-1.0
7	10067.07	10.10	2.0	.	12	9442.95	10.72	0.0	-2.0
18	10068.40	11.43	-0.5	-1.0	24	9443.72	11.49	-0.5	-0.5
4	10068.49	11.52	0.0	.	4	9444.95	12.72	0.0	.
11	10074.60	17.64	-0.5	-2.0	18	9446.21	13.98	-0.5	-1.0
17	10074.72	17.75	-1.0	-1.0	11	9449.48	17.25	-0.5	-2.0
16	10074.83	17.87	3.0	-2.0	17	9450.38	18.15	-1.0	-1.0
8	10085.26	28.30	3.0	.	8	9454.71	22.48	3.0	.
10	10086.65	29.68	-1.0	-2.0	10	9456.91	24.68	-1.0	-2.0
3	10091.90	34.93	-0.5	.	3	9463.66	31.43	-0.5	.
9	10112.86	55.89	-2.0	-2.0	9	9471.80	39.57	-2.0	-2.0
2	10131.55	74.59	-1.0	.	2	9494.66	62.43	-1.0	.
1	10193.29	136.32	-2.0	.	1	9541.55	109.32	-2.0	.

BDI - Battelle developmental inventory (screening version) measured at 4-years follow-up.

*Maternal schooling - Difference between linear model (bold line) and the model with lowest deviance (best model fit; first line in table) was not strong (p-value = 0.794).

**Paternal schooling - Difference between linear model (bold line) and the model with lowest deviance (best model fit; first line in table) was not strong (p-value = 0.224).

Table S5. Cutoff values of the predicted score of child development vulnerability (based on maternal and paternal schooling), and their test properties for classification of children below 10th percentile of BDI in 2015 Pelotas Birth Cohort.

**Test properties**	**Cutoff values**				
	**-0.029**	**-0.025**	**-0.020**	**-0.013**	**-0.008**
Sensitivity	59.6%	59.6%	60.8%	61.1%	64.9%
Especificity	59.2%	59.2%	58.9%	58.5%	54.7%
Positive predictive value	13.9%	13.9%	14.1%	14.0%	13.7%
Negative predictive value	93.0%	93.0%	93.1%	93.1%	93.4%
Percentage of positives in the cohort	42.7%	42.7%	43.1%	43.5%	46.8%
Correctly classified	59.2%	59.2%	59.1%	58.7%	55.4%

Table S6. Results of fractional polynomial analysis examining the linearity of the association between maternal schooling (complete years) and standardised BDI, and between paternal schooling (complete years) and standardised BDI, in the 2004 Pelotas Birth Cohort. Deviances for 44 combinations of two exponential terms (out of a series of eight: -2; -1; -0.5; 0; 0.5; 1; 2; 3) used by the standard Stata's package are presented.

**Maternal schooling***					**Paternal schooling****				
Order in Stata's output	Deviance	Difference from the best model	Exponent	Exponent	Order in Stata's output	Deviance	Difference from the best model	Exponent	Exponent
44	10342.92	0.00	3.0	3.0	43	8250.96	0.00	3.0	2.0
43	10344.38	1.46	3.0	2.0	42	8251.34	0.38	2.0	2.0
42	10345.82	2.90	2.0	2.0	44	8251.59	0.64	3.0	3.0
35	10347.06	4.14	0.5	0.5	31	8252.18	1.22	0.5	0.0
32	10347.16	4.24	1.0	0.0	26	8252.24	1.28	0.5	-0.5
36	10347.19	4.27	1.0	0.5	35	8252.39	1.43	0.5	0.5
27	10347.26	4.34	1.0	-0.5	30	8252.40	1.44	0.0	0.0
39	10347.36	4.44	1.0	1.0	20	8252.53	1.57	0.5	-1.0
21	10347.44	4.52	1.0	-1.0	41	8252.59	1.63	3.0	1.0
31	10347.67	4.75	0.5	0.0	14	8252.61	1.66	1.0	-2.0
14	10347.75	4.83	1.0	-2.0	21	8252.68	1.73	1.0	-1.0
40	10347.81	4.89	2.0	1.0	27	8252.74	1.78	1.0	-0.5
41	10348.11	5.19	3.0	1.0	40	8252.79	1.83	2.0	1.0
**6**	**10348.18**	**5.26**	**1.0**	**.**	32	8252.80	1.85	1.0	0.0
26	10348.92	6.00	0.5	-0.5	36	8252.86	1.90	1.0	0.5
37	10349.02	6.10	2.0	0.5	**6**	**8252.87**	**1.92**	**1.0**	**.**
30	10349.88	6.96	0.0	0.0	39	8252.87	1.92	1.0	1.0
33	10350.37	7.44	2.0	0.0	13	8253.33	2.37	0.5	-2.0
20	10350.45	7.53	0.5	-1.0	25	8253.46	2.50	0.0	-0.5
38	10351.46	8.54	3.0	0.5	37	8254.16	3.21	2.0	0.5
28	10351.77	8.84	2.0	-0.5	19	8255.13	4.17	0.0	-1.0
13	10352.98	10.06	0.5	-2.0	38	8255.42	4.47	3.0	0.5
22	10353.05	10.13	2.0	-1.0	33	8255.98	5.02	2.0	0.0
25	10353.44	10.52	0.0	-0.5	24	8256.34	5.39	-0.5	-0.5
15	10354.73	11.81	2.0	-2.0	5	8257.82	6.87	0.5	.
34	10356.27	13.35	3.0	0.0	28	8257.99	7.04	2.0	-0.5
7	10356.00	14.08	2.0	.	12	8258.82	7.86	0.0	-2.0

19	10357.59	14.67	0.0	-1.0	22	8259.82	8.86	2.0	-1.0
5	10358.48	15.56	0.5	.	34	8260.22	9.27	3.0	0.0
24	10360.22	17.30	-0.5	-0.5	18	8260.46	9.50	-0.5	-1.0
29	10362.40	19.48	3.0	-0.5	15	8262.09	11.13	2.0	-2.0
12	10364.72	21.80	0.0	-2.0	7	8264.20	13.24	2.0	.
18	10368.08	25.16	-0.5	-1.0	29	8266.53	15.58	3.0	-0.5
23	10368.64	25.72	3.0	-1.0	17	8267.89	16.93	-1.0	-1.0
16	10376.79	33.86	3.0	-2.0	11	8269.43	18.48	-0.5	-2.0
17	10380.37	37.45	-1.0	-1.0	23	8272.78	21.83	3.0	-1.0
11	10382.21	39.29	-0.5	-2.0	4	8277.29	26.33	0.0	.
8	10384.60	41.67	3.0	.	16	8280.60	29.65	3.0	-2.0
4	10385.79	42.87	0.0	.	10	8284.27	33.32	-1.0	-2.0
10	10403.32	60.40	-1.0	-2.0	8	8287.22	36.26	3.0	.
3	10433.60	90.68	-0.5	.	3	8315.54	64.58	-0.5	.
9	10444.35	101.43	-2.0	-2.0	9	8316.96	66.00	-2.0	-2.0
2	10490.44	147.52	-1.0	.	2	8362.57	111.62	-1.0	.
1	10559.26	216.34	-2.0	.	1	8420.19	169.23	-2.0	.

BDI - Battelle developmental inventory (screening version) measured at 4-years follow-up.

*Maternal schooling - Difference between linear model (bold line) and the model with lowest deviance (best model fit; first line in table) was not strong (p-value = 0.154)

**Paternal schooling - Difference between linear model (bold line) and the model with lowest deviance (best model fit; first line in table) was not strong (p-value = 0.590)

Table S7. Post-hoc analysis adding two perinatal predictors (gestational age and birth weight). Final adjusted linear regression model including predictors strongly associated ($p \leq 0.05$) with standardised Battelle Developmental Inventory (screening version) at age 4 years in 2015 Pelotas Birth Cohort (N = 3280).

Predictor	Coefficient	95% CI	p-value*
Maternal schooling (years)	0.03	0.02 to 0.04	<0.001
Paternal schooling (years)	0.02	0.01 to 0.03	<0.001
Family income (minimum wage quintile)			0.012
≤1.0	0		
1.1 to 3.0	0.08	-0.03 to 0.18	
3.1 to 6.0	0.13	0.01 to 0.25	
6.1 to 10.0	0.29	0.12 to 0.45	
>10.0	0.12	-0.06 to 0.30	
Maternal skin colour *X* Maternal depressive symptoms	-0.05	-0.10 to -0.01	0.015
Alcohol in pregnancy *X* Maternal adverse childhood experiences	-0.05	-0.09 to -0.00	0.037
Gestational age (week)	0.02	0.00 to 0.04	0.015
Birth weight (100 g)	0.01	0.00 to 0.02	0.013

*Wald test

CI = Confidence Interval

Adjusted for child's age and sex

Table S8. Post-hoc analysis adding two perinatal predictors (gestational age and birth weight). Linear regression model including 5 principal components (with eigenvalues > 1) predicting standardised Battelle Developmental Inventory (screening version) at age 4 years in 2015 Pelotas Birth Cohort (N = 3280).

Predictor	Coefficient	95% CI	p-value*
Principal component 1	**0.12**	**0.11 to 0.14**	**<0.001**
Principal component 2	**0.07**	**0.05 to 0.10**	**<0.001**
Principal component 3	0.01	-0.02 to 0.04	0.456
Principal component 4	0.01	-0.02 to 0.04	0.457
Principal component 5	0.03	-0.00 to 0.06	0.094

*Wald test

CI = Confidence Interval

Adjusted for child's age and sex

Table S9. Post-hoc analysis adding two perinatal predictors (gestational age and birth weight). Proportion of variance explained by each of the five Principal Components and eigenvectors for 13 predictors within each Principal Component.

Predictor	Comp1 23% of variance	Comp2 12% of variance	Comp3 11% of variance	Comp4 9% of variance	Comp5 8% of variance
Maternal age (<20/20+)	0.19	0.03	**0.59**	0.26	-0.27
Maternal schooling (years)	**0.49**	0.01	0.06	0.20	0.11
Paternal schooling (years)	**0.46**	-0.00	0.06	0.17	0.15
Family income (minimum wage quintile)	**0.43**	-0.00	0.11	0.16	0.11
Maternal skin colour (white/non-white)	-0.27	0.00	-0.16	**0.58**	-0.08
Paternal skin colour (white/non-white)	-0.24	0.01	-0.19	**0.63**	-0.11
Number of children living with the mother (1/2/3/4+)	-0.21	0.07	**0.59**	0.06	**-0.32**
Mother smoked during pregnancy (no/yes)	-0.24	-0.14	**0.32**	-0.10	0.26
Maternal alcohol use during pregnancy (no/yes)	-0.09	0.01	0.14	0.28	**0.75**
Maternal Adverse Childhood Experiences (frequency – 1 to 9)	-0.22	-0.03	0.20	-0.09	0.28
Postnatal maternal depressive symptoms (low/moderate/significant)	-0.20	-0.07	0.24	-0.03	0.18
Gestational age (days)	-0.06	**0.69**	-0.05	-0.06	0.08
Birth weights (grams)	-0.00	**0.70**	0.06	-0.00	0.04

Box S1. Measurements details of the 27 potential predictors of childhood development at age 4 years, and operationalisation in conditional inference tree analysis.

Covariates	Type of measurement	Follow-up of 2015 Pelotas Birth Cohort in which it was collected	Operationalization in conditional inference tree analysis
Garbage accumulated in the neighborhood	Maternal report	Prenatal assessment	No/yes
Open sewer in the neighbourhood	Maternal report	Prenatal assessment	No/yes
Maternal drug use during pregnancy	Maternal report	Prenatal assessment	No/yes
Prenatal maternal depressive symptoms	Maternal report	Prenatal assessment	Edimburg scale score (0 to 30)
Maternal skin colour	Maternal report	Perinatal assessment	White/black/brown/Asian/Indigenous
Paternal skin colour	Maternal report	Perinatal assessment	White/black/brown/Asian/Indigenous
Maternal alcohol use during pregnancy	Maternal report	Perinatal assessment	No/yes
Mother smoked during pregnancy	Maternal report	Perinatal assessment	No/yes
Mother's partner smokes at perinatal	Maternal report	Perinatal assessment	No/yes
People per bedroom in the house	Maternal report	Perinatal assessment	≤ 2; 2.1 – 3.0; 3.1 – 4.0; > 4
Number of children living with the mother	Maternal report	Perinatal assessment	0; 1; 2; 3; 4 or more
Planned pregnancy	Maternal report	Perinatal assessment	No/yes
Father's support level during pregnancy	Maternal report	Perinatal assessment	Little or none/medium/much
Mother living with partner at birth	Maternal report	Perinatal assessment	No/yes
Mother worked during pregnancy	Maternal report	Perinatal assessment	No/yes
Father working at perinatal	Maternal report	Perinatal assessment	No/yes
Maternal diabetes during pregnancy	Maternal report	Perinatal assessment	No/yes
Maternal hipertension during pregnancy	Maternal report	Perinatal assessment	No/yes
Maternal anemia during pregnancy	Maternal report	Perinatal assessment	No/yes
Maternal age at birth	Maternal report	Perinatal assessment	complete years
Maternal schooling	Maternal report	Perinatal assessment	complete years
Paternal schooling	Maternal report	Perinatal assessment	complete years
Family income at birth	Maternal report	Perinatal assessment	Minimum wage (continuum)
Postnatal maternal depressive symptoms	Maternal report	3-month assessment	Edimburg scale score (0 to 30)
Couple relationship characterized by criticism	Maternal report	3-month assessment	Low/medium/high
Neighborhood violence	Maternal report	48-month assessment	Low/medium/high
Maternal Adverse Childhood Experiences	Maternal report	48-month assessment	Score (0 to 9)

Box S2. 11 potential predictors of childhood development for which interaction terms (among all them) were included in exploratory linear regression analysis. The same set of potential predictors were used in Principal Component Analysis (without interaction terms).

Predictor	Operacionalisation in linear regression and PCA analyses	Follow-up of 2015 Pelotas Birth Cohort in which it was collected
Maternal age	<20/20+	Perinatal assessment
Maternal schooling	complete years	Perinatal assessment
Paternal schooling	complete years	Perinatal assessment
Family income	minimum wage quintile	Perinatal assessment
Maternal skin colour	white/non-white	Perinatal assessment
Paternal skin colour	white/non-white	Perinatal assessment
Number of children living with the mother	1; 2; 3; 4+	Perinatal assessment
Mother smoked during pregnancy	no/yes	Perinatal assessment
Maternal alcohol use during pregnancy	no/yes	Perinatal assessment
Postnatal maternal depressive symptoms	low(0-9)/moderate(10-12)/significant(13+)	3-month assessment
Maternal Adverse Childhood Experiences	score (0 to 9)	48-month assessment

Box S3. Theoretical coverage and focus that would be achieved by a home visiting programme if targeting were based on the presented predictors and cutoff points. Data from 2015 Pelotas Birth Cohort Study (n = 3603).

A	B	C*	D**	E***	F	G	H	I
**Variable**	**Cutoff**	**% of 2015 cohort below the cutoff**	**% of all children of the lowest BDI decile in this subgroup (coverage)**	**% of children of this subgroup in the lowest BDI decile (focus)**	**Actual PIM coverage starting during pregnancy in this subgroup (%)**	**Actual PIM focus starting during pregnancy in this subgroup (%)**	**Actual PIM coverage starting after birth in this subgroup (%)**	**Actual PIM focus starting after birth in this subgroup (%)**
Maternal schooling (years)	<6	15	24	16	9	34	31	30
Paternal schooling (years)	<5	12	19	15	6	19	28	22
Maternal and Paternal schooling (years), respectively	<6 and <5	6	10	20	9	11	34	11
Maternal and Paternal schooling (years), respectively	<10 and <10	30	44	15	7	51	25	47
Birth weight (grams)	<2500	10	14	16	-	-	20	12
Gestational age (weeks)	<37	14	19	14	-	-	20	18

BDI: Battelle Developmental Inventory
PIM: Primeira Infância Melhor Home Visiting Programme
*Column C provides an estimate of the required size of the program to cover this subgroup.
**Column D provides theoretical coverage of a programme reaching all children in the subgroup.
***Column E provides theoretical focus of a programme reaching all the children in the subgroup.

CODE USED IN ANALYSES

Code used in R version 4.1.0

```
# Libraries
install.packages("ggplot2")
install.packages("haven")
install.packages("matrixStats")
install.packages("ggridges")
install.packages("party")
install.packages("partykit")
install.packages("Rmisc")
install.packages("rpart")
install.packages("rpart.plot")
install.packages("tidyverse")

suppressMessages(library(ggplot2))
suppressMessages(library(haven))
suppressMessages(library(matrixStats))
suppressMessages(library(ggridges))
suppressMessages(library(party))
suppressMessages(library(partykit))
suppressMessages(library(Rmisc))
suppressMessages(library(rpart))
suppressMessages(library(rpart.plot))
suppressMessages(library(tidyverse))

#Working directory
setwd("C:/Tese/Artigo 3/Project_test_R/ECD_TREE_C2015")

# Dataset
ECD_TREE_C2015 <- read.csv("ECD_TREE_C2015_20221219.csv", header = TRUE, sep
    = ",")
ECD_TREE_C2015 <-
    ECD_TREE_C2015[!is.na(ECD_TREE_C2015$zbattelle_adjidadesex), ]

# Tranforming variables, characters to factors
ECD_TREE_C2015$sex = factor(ECD_TREE_C2015$sex, ordered = FALSE, levels =
    c("Masculino", "Feminino"), labels = c("Masculino", "Feminino"))
ECD_TREE_C2015$maternal_skin_color = factor(ECD_TREE_C2015$maternal_skin_color,
    ordered = FALSE, levels = c("Branca", "Preta", "Amarela", "Morena/parda",
    "Indigena"), labels = c("Branca", "Preta", "Amarela", "Morena/parda", "Indigena"))
ECD_TREE_C2015$paternal_skin_color = factor(ECD_TREE_C2015$paternal_skin_color,
    ordered = FALSE, levels = c("Branca", "Negra", "Morena/parda", "Amarela",
    "Indigena"), labels = c("Branca", "Preta", "Morena/parda", "Amarela", "Indigena"))
ECD_TREE_C2015$neighborhood_violence =
    factor(ECD_TREE_C2015$neighborhood_violence, ordered = TRUE, levels =
    c("Low", "Median", "High"), labels = c("Low", "Median", "High"))
ECD_TREE_C2015$garbage_accumulated =
    factor(ECD_TREE_C2015$garbage_accumulated, ordered = FALSE, levels =
```

```
c("Nao", "Sim"), labels = c("Nao", "Sim"))
ECD_TREE_C2015$open_sewer = factor(ECD_TREE_C2015$open_sewer, ordered =
    FALSE, levels = c("Nao", "Sim"), labels = c("Nao", "Sim"))
ECD_TREE_C2015$drug_pregnancy = factor(ECD_TREE_C2015$drug_pregnancy,
    ordered = FALSE, levels = c("Nao", "Sim"), labels = c("Nao", "Sim"))
ECD_TREE_C2015$alcohol_pregnancy = factor(ECD_TREE_C2015$alcohol_pregnancy,
    ordered = FALSE, levels = c("Nao", "Sim"), labels = c("Nao", "Sim"))
ECD_TREE_C2015$smoking_pregnancy = factor(ECD_TREE_C2015$smoking_pregnancy,
    ordered = FALSE, levels = c("Nao", "Sim"), labels = c("Nao", "Sim"))
ECD_TREE_C2015$smoking_partner = factor(ECD_TREE_C2015$smoking_partner,
    ordered = FALSE, levels = c("Nao", "Sim"), labels = c("Nao", "Sim"))
ECD_TREE_C2015$density_in_house = factor(ECD_TREE_C2015$density_in_house,
    ordered = TRUE, levels = c("Ate 2", ">2 ate 3", ">3 ate 4", ">4"), labels = c("Ate 2", ">2
    ate 3", ">3 ate 4", ">4"))
ECD_TREE_C2015$number_children = factor(ECD_TREE_C2015$number_children,
    ordered = TRUE, levels = c("0", "1", "2", "3", "4 ou mais"), labels = c("0", "1", "2", "3",
    "4 ou mais"))
ECD_TREE_C2015$planned_pregnancy = factor(ECD_TREE_C2015$planned_pregnancy,
    ordered = FALSE, levels = c("Planejou", "Sem querer ou mais ou menos"), labels =
    c("Planejou", "Sem querer ou mais ou menos"))
ECD_TREE_C2015$paternal_support = factor(ECD_TREE_C2015$paternal_support,
    ordered = TRUE, levels = c("Low", "Median", "High"), labels = c("Low", "Median",
    "High"))
ECD_TREE_C2015$couple_criticism = factor(ECD_TREE_C2015$couple_criticism, ordered
    = TRUE, levels = c("Low criticism", "Median criticism", "High criticism"), labels =
    c("Low", "Median", "High"))
ECD_TREE_C2015$mother_with_partner =
    factor(ECD_TREE_C2015$mother_with_partner, ordered = FALSE, levels = c("Nao",
    "Sim"), labels = c("Nao", "Sim"))
ECD_TREE_C2015$worked_in_pregnancy =
    factor(ECD_TREE_C2015$worked_in_pregnancy, ordered = FALSE, levels = c("Nao",
    "Sim"), labels = c("Nao", "Sim"))
ECD_TREE_C2015$father_working = factor(ECD_TREE_C2015$father_working, ordered =
    FALSE, levels = c("no", "yes"), labels = c("no", "yes"))
ECD_TREE_C2015$diabetes_pregnancy = factor(ECD_TREE_C2015$diabetes_pregnancy,
    ordered = FALSE, levels = c("Nao", "Sim"), labels = c("Nao", "Sim"))
ECD_TREE_C2015$has_pregnancy = factor(ECD_TREE_C2015$has_pregnancy, ordered
    = FALSE, levels = c("Nao", "Sim"), labels = c("Nao", "Sim"))
ECD_TREE_C2015$maternal_anemia = factor(ECD_TREE_C2015$maternal_anemia,
    ordered = FALSE, levels = c("Nao", "Sim"), labels = c("Nao", "Sim"))
ECD_TREE_C2015$f_battelle_total_exc_cat =
    factor(ECD_TREE_C2015$f_battelle_total_exc_cat, ordered = FALSE, levels =
    c("Average development", "Suspected developmental delay"), labels = c("Average
    development", "Suspected developmental delay"))

# Tranforming variables, characters to integer
ECD_TREE_C2015$maternal_age = as.numeric(ECD_TREE_C2015$maternal_age)
ECD_TREE_C2015$depression_simptons_antenatal =
    as.numeric(ECD_TREE_C2015$depression_simptons_antenatal)
ECD_TREE_C2015$maternal_ACEs = as.numeric(ECD_TREE_C2015$maternal_ACEs)
ECD_TREE_C2015$sispim_gestacao_numeric =
    as.numeric(ECD_TREE_C2015$sispim_gestacao_numeric)
```

```
#Generating a tree adjusted for sex and age of the child
set.seed(2)
ctree.model <- ctree(zbattelle_adjidadesex ~ maternal_schooling + paternal_schooling +
        maternal_age +
              maternal_skin_color + paternal_skin_color + neighborhood_violence +
              family_income_minimum_wage + garbage_accumulated + open_sewer +
              drug_pregnancy + alcohol_pregnancy + smoking_pregnancy +
        smoking_partner +
              depression_simptons_antenatal + depression_simptons_postnatal +
              density_in_house + number_children + planned_pregnancy +
              paternal_support + couple_criticism + maternal_ACEs + mother_with_partner
        +
              worked_in_pregnancy + father_working + diabetes_pregnancy +
        has_pregnancy +
              maternal_anemia, minbucket = 50, alpha = 0.05, data = ECD_TREE_C2015,
             maxsurrogate = 5)
ctree.model

# Figure
plot(ctree.model, gp = gpar(fontsize = 10))

# Adding nodes
data.predict <- ECD_TREE_C2015
set.seed(2)
data.predict$node <- predict(ctree.model, newdata = ECD_TREE_C2015)
data.predict <- data.predict %>%
  mutate(node = factor(node))

# Distribution
# Graph density, overlapping
ggplot(data.predict) +
  aes(x = zbattelle_adjidadesex, color = node) +
  geom_density() +
  theme_light() +
  theme(legend.position = "bottom")
# Graph density, separated curves
data.predict <- data.predict %>%
  mutate(node = factor(node,
              levels = c(-0.478750685538785,
                   -0.261385128696439,
                   -0.14647924454164,
                   0.0362240135601695,
                   0.264545930767598,
                   0.309870430239811),
              labels = c("Group 1 (n = 214):\n-0.48 (-0.63,-0.33)",
                   "Group 2 (n = 337):\n-0.26 (-0.35,-0.17)",
                   "Group 3 (n = 927):\n-0.15 (-0.21,-0.08)",
                   "Group 4 (n = 1180):\n0.04 (-0.02,0.09)",
                   "Group 5 (n = 204):\n0.26 (0.14,0.39)",
                   "Group 6 (n = 741):\n0.31 (0.25,0.38)")))

# Ridgeline plot
ggplot(data.predict) +
```

```
  aes(x = zbattelle_adjidadesex, color = node, fill = node, y = node) +
  geom_vline(xintercept = 0, color = "gray70", linewidth = 0.4, linetype = "dashed") +
  geom_density_ridges(size = 0.8, key_glyph = draw_key_path, alpha = 0.2,
                      show.legend = FALSE, scale = 2) +
  theme_light() +
  theme(legend.position = "bottom",
        panel.grid.minor = element_blank(),
        panel.grid.major.y = element_blank(),
        panel.grid.major.x = element_line(color = "gray92"),
        panel.border = element_blank(),
        axis.line = element_line(color = "gray70", linewidth = 0.4),
        axis.ticks = element_line(color = "gray70", linewidth = 0.4),
        legend.key.width = unit(30, "points"),
        axis.title = element_text(size = text.size*1.2, color = text.color),
        axis.text.x = element_text(size = text.size, color = text.color),
        axis.text.y = element_text(size = text.size, color = text.color, vjust = 0),
        legend.text = element_text(size = text.size*0.9, color = text.color),
        legend.title = element_text(size = text.size*0.9, color = text.color),
        plot.margin = margin(10,10,10,10)) +
  labs(x = "Battelle Developmental Inventory (screening version) â□□ standardized score",
       y = "Groups generated by decision tree (mean and 95% CI)") +
  coord_cartesian(xlim = c(-4, 2)) +
  scale_x_continuous(breaks = seq(-6.5, 2, 0.5), expand = expansion(add = 0)) +
  scale_y_discrete(expand = expansion(add = c(0,2.1))) +
  scale_colour_manual(values = c("#F95C62", "#FA8267", "#FAC6C7",
                                 "#9FCAE5", "#15679A", "#03245E")) +
  scale_fill_manual(values = c("#F95C62", "#FA8267", "#FAC6C7",
                               "#9FCAE5", "#15679A", "#03245E"),
                    guide = "none") +
  guides(color = guide_legend(nrow = 2, byrow = TRUE,
                              override.aes = list(alpha = 1)))

ggsave("ridgeline_plot.png", height = 6, width = 9)


# Confidence intervals
data.predict %>%
  group_by(node) %>%
  dplyr::summarise(mean = mean(zbattelle_adjidadesex),
                   ll = CI(zbattelle_adjidadesex)[3],
                   ul = CI(zbattelle_adjidadesex)[1])

#Finding surrogate splits when covariates are missing
#node 1
sur11 <- nodeapply(ctree.model, ids = 1, function(n) n$surrogates[[1]])
character_split(sur11[[1]], model.frame(ctree.model))

sur12 <- nodeapply(ctree.model, ids = 1, function(n) n$surrogates[[2]])
character_split(sur12[[1]], model.frame(ctree.model))

sur13 <- nodeapply(ctree.model, ids = 1, function(n) n$surrogates[[3]])
character_split(sur13[[1]], model.frame(ctree.model))
```

```
sur14 <- nodeapply(ctree.model, ids = 1, function(n) n$surrogates[[4]])
character_split(sur14[[1]], model.frame(ctree.model))

sur15 <- nodeapply(ctree.model, ids = 1, function(n) n$surrogates[[5]])
character_split(sur15[[1]], model.frame(ctree.model))

#node 2
sur21 <- nodeapply(ctree.model, ids = 2, function(n) n$surrogates[[1]])
character_split(sur21[[1]], model.frame(ctree.model))

sur22 <- nodeapply(ctree.model, ids = 2, function(n) n$surrogates[[2]])
character_split(sur22[[1]], model.frame(ctree.model))

sur23 <- nodeapply(ctree.model, ids = 2, function(n) n$surrogates[[3]])
character_split(sur23[[1]], model.frame(ctree.model))

sur24 <- nodeapply(ctree.model, ids = 2, function(n) n$surrogates[[4]])
character_split(sur24[[1]], model.frame(ctree.model))

sur25 <- nodeapply(ctree.model, ids = 2, function(n) n$surrogates[[5]])
character_split(sur25[[1]], model.frame(ctree.model))

#node 3
sur31 <- nodeapply(ctree.model, ids = 3, function(n) n$surrogates[[1]])
character_split(sur31[[1]], model.frame(ctree.model))

sur32 <- nodeapply(ctree.model, ids = 3, function(n) n$surrogates[[2]])
character_split(sur32[[1]], model.frame(ctree.model))

sur33 <- nodeapply(ctree.model, ids = 3, function(n) n$surrogates[[3]])
character_split(sur33[[1]], model.frame(ctree.model))

sur34 <- nodeapply(ctree.model, ids = 3, function(n) n$surrogates[[4]])
character_split(sur34[[1]], model.frame(ctree.model))

sur35 <- nodeapply(ctree.model, ids = 3, function(n) n$surrogates[[5]])
character_split(sur35[[1]], model.frame(ctree.model))

#node 7
sur71 <- nodeapply(ctree.model, ids = 7, function(n) n$surrogates[[1]])
character_split(sur71[[1]], model.frame(ctree.model))

sur72 <- nodeapply(ctree.model, ids = 7, function(n) n$surrogates[[2]])
character_split(sur72[[1]], model.frame(ctree.model))

sur73 <- nodeapply(ctree.model, ids = 7, function(n) n$surrogates[[3]])
character_split(sur73[[1]], model.frame(ctree.model))

sur74 <- nodeapply(ctree.model, ids = 7, function(n) n$surrogates[[4]])
character_split(sur74[[1]], model.frame(ctree.model))

sur75 <- nodeapply(ctree.model, ids = 7, function(n) n$surrogates[[5]])
character_split(sur75[[1]], model.frame(ctree.model))
```

```
#node 8
sur81 <- nodeapply(ctree.model, ids = 8, function(n) n$surrogates[[1]])
character_split(sur81[[1]], model.frame(ctree.model))

sur82 <- nodeapply(ctree.model, ids = 8, function(n) n$surrogates[[2]])
character_split(sur82[[1]], model.frame(ctree.model))

sur83 <- nodeapply(ctree.model, ids = 8, function(n) n$surrogates[[3]])
character_split(sur83[[1]], model.frame(ctree.model))

sur84 <- nodeapply(ctree.model, ids = 8, function(n) n$surrogates[[4]])
character_split(sur84[[1]], model.frame(ctree.model))

sur85 <- nodeapply(ctree.model, ids = 8, function(n) n$surrogates[[5]])
character_split(sur85[[1]], model.frame(ctree.model))

# R-squared of tree final nodes
data.predict$node_categorical <- ifelse(data.predict$node == -0.478750685538785, 1,
                                 ifelse(data.predict$node == -0.261385128696439, 2,
                                 ifelse(data.predict$node == -0.14647924454164, 3,
                                 ifelse(data.predict$node == 0.0362240135601695, 4,
                                 ifelse(data.predict$node == 0.264545930767598, 5,
                                 ifelse(data.predict$node == 0.309870430239811, 6, 0))))))
lm(zbattelle_adjidadesex ~ node_categorical, data = data.predict) %>%
  summary()

data.predict$node_categorical_factor = factor(data.predict$node_categorical, ordered =
        FALSE, levels = c("1", "2", "3", "4", "5", "6"), labels = c("1", "2", "3", "4", "5", "6"))
lm(zbattelle_adjidadesex ~ node_categorical_factor, data = data.predict) %>%
  summary()

# Low ECD score in final nodes
node1 <- subset(data.predict, node == "-0.478750685538785")
crosstab1 <- table(node1$f_battelle_total_exc_cat)
prop.table(crosstab1)

node2 <- subset(data.predict, node == "-0.261385128696439")
crosstab2 <- table(node2$f_battelle_total_exc_cat)
prop.table(crosstab2)

node3 <- subset(data.predict, node == "-0.14647924454164")
crosstab3 <- table(node3$f_battelle_total_exc_cat)
prop.table(crosstab3)

node4 <- subset(data.predict, node == "0.0362240135601695")
crosstab4 <- table(node4$f_battelle_total_exc_cat)
prop.table(crosstab4)

node5 <- subset(data.predict, node == "0.264545930767598")
crosstab5 <- table(node5$f_battelle_total_exc_cat)
prop.table(crosstab5)
```

```
node6 <- subset(data.predict, node == "0.309870430239811")
crosstab6 <- table(node6$f_battelle_total_exc_cat)
prop.table(crosstab6)



# ---------------------------------------------------------------------------------------- #
## Sensitivity test - 3461 children with all 27 variables, excluding children receiving PIM
	during pregnancy

# Excluding children receiving PIM during pregnancy
ECD_TREE_C2015 <-
	ECD_TREE_C2015[!is.na(ECD_TREE_C2015$zbattelle_adjidadesex), ]
ECD_TREE_C2015_sem_pim <-
	ECD_TREE_C2015[ECD_TREE_C2015$sispim_gestacao_numeric!=1, ]

#Generating the tree
set.seed(2)
ctree.model <- ctree(zbattelle_adjidadesex ~ maternal_age + maternal_skin_color +
	paternal_skin_color +
		neighborhood_violence + maternal_schooling + paternal_schooling +
		family_income_minimum_wage + garbage_accumulated + open_sewer +
		drug_pregnancy + alcohol_pregnancy + smoking_pregnancy +
	smoking_partner +
		depression_simptons_antenatal + depression_simptons_postnatal +
		density_in_house + number_children + planned_pregnancy +
		paternal_support + couple_criticism + maternal_ACEs + mother_with_partner
	+
		worked_in_pregnancy + father_working + diabetes_pregnancy +
	has_pregnancy +
		maternal_anemia, minbucket = 50, alpha = 0.05, data =
	ECD_TREE_C2015_sem_pim,
		maxsurrogate = 5)
ctree.model

# Figure
plot(ctree.model, gp = gpar(fontsize = 10))

# Adding nodes
data.predict <- ECD_TREE_C2015_sem_pim
set.seed(2)
data.predict$node <- predict(ctree.model, newdata = ECD_TREE_C2015_sem_pim)
data.predict <- data.predict %>%
  mutate(node = factor(node))

# Distribution
ggplot(data.predict) +
  aes(x = zbattelle_adjidadesex, color = node) +
  geom_density() +
  theme_light() +
  theme(legend.position = "bottom")

# Confidence intervals
```

```
data.predict %>%
  group_by(node) %>%
  dplyr::summarise(mean = mean(zbattelle_adjidadesex),
                   ll = CI(zbattelle_adjidadesex)[3],
                   ul = CI(zbattelle_adjidadesex)[1])



# ------------------------------------------------------------------------------------------- #
#External validation with 2004 Pelotas cohort

# Dataset
ECD_TREE_C2004 <- read.csv("ECD_TREE_C2004_20221219.csv", header = TRUE, sep
        = ",")
ECD_TREE_C2004 <-
        ECD_TREE_C2004[!is.na(ECD_TREE_C2004$zbattelle_adjidadesex), ]

# Tranforming variables, characters to factors
ECD_TREE_C2004$sex = factor(ECD_TREE_C2004$sex, ordered = FALSE, levels =
        c("Masculino", "Feminino"), labels = c("Masculino", "Feminino"))
ECD_TREE_C2004$maternal_skin_color = factor(ECD_TREE_C2004$maternal_skin_color,
        ordered = FALSE, levels = c("branca", "preta", "amarela", "morena/parda", "indigena"),
        labels = c("Branca", "Preta", "Amarela", "Morena/parda", "Indigena"))
ECD_TREE_C2004$paternal_skin_color = factor(ECD_TREE_C2004$paternal_skin_color,
        ordered = FALSE, levels = c("branca", "negra", "morena ou parda", "amarela ou
        asiatica", "indigena"), labels = c("Branca", "Preta", "Morena/parda", "Amarela",
        "Indigena"))
ECD_TREE_C2004$neighborhood_violence =
        factor(ECD_TREE_C2004$neighborhood_violence, ordered = TRUE, levels =
        c("Low", "Median", "High"), labels = c("Low", "Median", "High"))
ECD_TREE_C2004$garbage_accumulated =
        factor(ECD_TREE_C2004$garbage_accumulated, ordered = FALSE, levels =
        c("Nao", "Sim"), labels = c("Nao", "Sim"))
ECD_TREE_C2004$open_sewer = factor(ECD_TREE_C2004$open_sewer, ordered =
        FALSE, levels = c("Nao", "Sim"), labels = c("Nao", "Sim"))
ECD_TREE_C2004$drug_pregnancy = factor(ECD_TREE_C2004$drug_pregnancy,
        ordered = FALSE, levels = c("Nao", "Sim"), labels = c("Nao", "Sim"))
ECD_TREE_C2004$alcohol_pregnancy = factor(ECD_TREE_C2004$alcohol_pregnancy,
        ordered = FALSE, levels = c("nao", "sim"), labels = c("Nao", "Sim"))
ECD_TREE_C2004$smoking_pregnancy = factor(ECD_TREE_C2004$smoking_pregnancy,
        ordered = FALSE, levels = c("nao", "sim"), labels = c("Nao", "Sim"))
ECD_TREE_C2004$smoking_partner = factor(ECD_TREE_C2004$smoking_partner,
        ordered = FALSE, levels = c("nao", "sim"), labels = c("Nao", "Sim"))
ECD_TREE_C2004$density_in_house = factor(ECD_TREE_C2004$density_in_house,
        ordered = TRUE, levels = c("Ate 2", ">2 ate 3", ">3 ate 4", ">4"), labels = c("Ate 2", ">2
        ate 3", ">3 ate 4", ">4"))
ECD_TREE_C2004$number_children = factor(ECD_TREE_C2004$number_children,
        ordered = TRUE, levels = c("0", "1", "2", "3", "4 ou mais"), labels = c("0", "1", "2", "3",
        "4 ou mais"))
ECD_TREE_C2004$planned_pregnancy = factor(ECD_TREE_C2004$planned_pregnancy,
        ordered = FALSE, levels = c("Planejou", "Sem querer ou mais ou menos"), labels =
        c("Planejou", "Sem querer ou mais ou menos"))
ECD_TREE_C2004$paternal_support = factor(ECD_TREE_C2004$paternal_support,
```

```
        ordered = TRUE, levels = c("Low", "Median", "High"), labels = c("Low", "Median",
        "High"))
ECD_TREE_C2004$couple_criticism = factor(ECD_TREE_C2004$couple_criticism, ordered
        = TRUE, levels = c("Low criticism", "Median criticism", "High criticism"), labels =
        c("Low", "Median", "High"))
ECD_TREE_C2004$mother_with_partner =
        factor(ECD_TREE_C2004$mother_with_partner, ordered = FALSE, levels = c("nao",
        "sim"), labels = c("Nao", "Sim"))
ECD_TREE_C2004$worked_in_pregnancy =
        factor(ECD_TREE_C2004$worked_in_pregnancy, ordered = FALSE, levels = c("nao",
        "sim"), labels = c("Nao", "Sim"))
ECD_TREE_C2004$father_working = factor(ECD_TREE_C2004$father_working, ordered =
        FALSE, levels = c("no", "yes"), labels = c("no", "yes"))
ECD_TREE_C2004$diabetes_pregnancy = factor(ECD_TREE_C2004$diabetes_pregnancy,
        ordered = FALSE, levels = c("nao", "sim"), labels = c("Nao", "Sim"))
ECD_TREE_C2004$has_pregnancy = factor(ECD_TREE_C2004$has_pregnancy, ordered
        = FALSE, levels = c("Nao", "Sim"), labels = c("Nao", "Sim"))
ECD_TREE_C2004$maternal_anemia = factor(ECD_TREE_C2004$maternal_anemia,
        ordered = FALSE, levels = c("Nao", "Sim"), labels = c("Nao", "Sim"))
ECD_TREE_C2004$f_battelle_total_exc_cat =
        factor(ECD_TREE_C2004$f_battelle_total_exc_cat, ordered = FALSE, levels =
        c("Average development", "Suspected developmental delay"), labels = c("Average
        development", "Suspected developmental delay"))
# TranformaÃ§Ã£o das variÃ¡veis characters para integer
ECD_TREE_C2004$maternal_age = as.numeric(ECD_TREE_C2004$maternal_age)
ECD_TREE_C2004$depression_simptons_antenatal =
        as.numeric(ECD_TREE_C2004$depression_simptons_antenatal)
ECD_TREE_C2004$maternal_ACEs = as.numeric(ECD_TREE_C2004$maternal_ACEs)
ECD_TREE_C2004$sispim_gestacao_numeric =
        as.numeric(ECD_TREE_C2004$sispim_gestacao_numeric)

# Adding nodes
data.predict <- ECD_TREE_C2004
set.seed(2)
data.predict$node <- predict(ctree.model, newdata = ECD_TREE_C2004)
data.predict <- data.predict %>%
  mutate(node = factor(node))

# Distribution
ggplot(data.predict) +
  aes(x = zbattelle_adjidadesex, color = node) +
  geom_density() +
  theme_light() +
  theme(legend.position = "bottom")

# Confidence intervals
data.predict %>%
  group_by(node) %>%
  dplyr::summarise(mean = mean(zbattelle_adjidadesex),
            ll = CI(zbattelle_adjidadesex)[3],
            ul = CI(zbattelle_adjidadesex)[1])

# R-squared of the tree
```

```
data.predict$node_categorical <- ifelse(data.predict$node == -0.478750685538785, 1,
                                 ifelse(data.predict$node == -0.261385128696439, 2,
                                 ifelse(data.predict$node == -0.14647924454164, 3,
                                 ifelse(data.predict$node == 0.0362240135601695, 4,
                                 ifelse(data.predict$node == 0.264545930767598, 5,
                                 ifelse(data.predict$node == 0.309870430239811, 6, 0))))))
lm(zbattelle_adjidadesex ~ node_categorical, data = data.predict) %>%
  summary()

data.predict$node_categorical_factor = factor(data.predict$node_categorical, ordered =
      FALSE,
                                    levels = c("1", "2", "3", "4", "5", "6"),
                                    labels = c("1", "2", "3", "4", "5", "6"))
lm(zbattelle_adjidadesex ~ node_categorical_factor, data = data.predict) %>%
  summary()

# Low ECD score in final nodes
node1 <- subset(data.predict, node == "-0.478750685538785")
crosstab1 <- table(node1$f_battelle_total_exc_cat)
prop.table(crosstab1)

node2 <- subset(data.predict, node == "-0.261385128696439")
crosstab2 <- table(node2$f_battelle_total_exc_cat)
prop.table(crosstab2)

node3 <- subset(data.predict, node == "-0.14647924454164")
crosstab3 <- table(node3$f_battelle_total_exc_cat)
prop.table(crosstab3)

node4 <- subset(data.predict, node == "0.0362240135601695")
crosstab4 <- table(node4$f_battelle_total_exc_cat)
prop.table(crosstab4)

node5 <- subset(data.predict, node == "0.264545930767598")
crosstab5 <- table(node5$f_battelle_total_exc_cat)
prop.table(crosstab5)

node6 <- subset(data.predict, node == "0.309870430239811")
crosstab6 <- table(node6$f_battelle_total_exc_cat)
prop.table(crosstab6)




# ------------------------------------------------------------------------------------------- #
#Sensitivity Analysis in 2015 cohort adding two predictors: gestational age and birthweight

# Dataset
ECD_TREE_C2015 <- read.csv("ECD_TREE_C2015_20231203.csv", header = TRUE, sep
      = ",")
ECD_TREE_C2015 <-
      ECD_TREE_C2015[!is.na(ECD_TREE_C2015$zbattelle_adjidadesex), ]
```

```
# Tranforming variables, characters to factors
ECD_TREE_C2015$sex = factor(ECD_TREE_C2015$sex, ordered = FALSE, levels =
    c("Masculino", "Feminino"), labels = c("Masculino", "Feminino"))
ECD_TREE_C2015$maternal_skin_color = factor(ECD_TREE_C2015$maternal_skin_color,
    ordered = FALSE, levels = c("Branca", "Preta", "Amarela", "Morena/parda",
    "Indigena"), labels = c("Branca", "Preta", "Amarela", "Morena/parda", "Indigena"))
ECD_TREE_C2015$paternal_skin_color = factor(ECD_TREE_C2015$paternal_skin_color,
    ordered = FALSE, levels = c("Branca", "Negra", "Morena/parda", "Amarela",
    "Indigena"), labels = c("Branca", "Preta", "Morena/parda", "Amarela", "Indigena"))
ECD_TREE_C2015$neighborhood_violence =
    factor(ECD_TREE_C2015$neighborhood_violence, ordered = TRUE, levels =
    c("Low", "Median", "High"), labels = c("Low", "Median", "High"))
ECD_TREE_C2015$garbage_accumulated =
    factor(ECD_TREE_C2015$garbage_accumulated, ordered = FALSE, levels =
    c("Nao", "Sim"), labels = c("Nao", "Sim"))
ECD_TREE_C2015$open_sewer = factor(ECD_TREE_C2015$open_sewer, ordered =
    FALSE, levels = c("Nao", "Sim"), labels = c("Nao", "Sim"))
ECD_TREE_C2015$drug_pregnancy = factor(ECD_TREE_C2015$drug_pregnancy,
    ordered = FALSE, levels = c("Nao", "Sim"), labels = c("Nao", "Sim"))
ECD_TREE_C2015$alcohol_pregnancy = factor(ECD_TREE_C2015$alcohol_pregnancy,
    ordered = FALSE, levels = c("Nao", "Sim"), labels = c("Nao", "Sim"))
ECD_TREE_C2015$smoking_pregnancy = factor(ECD_TREE_C2015$smoking_pregnancy,
    ordered = FALSE, levels = c("Nao", "Sim"), labels = c("Nao", "Sim"))
ECD_TREE_C2015$smoking_partner = factor(ECD_TREE_C2015$smoking_partner,
    ordered = FALSE, levels = c("Nao", "Sim"), labels = c("Nao", "Sim"))
ECD_TREE_C2015$density_in_house = factor(ECD_TREE_C2015$density_in_house,
    ordered = TRUE, levels = c("Ate 2", ">2 ate 3", ">3 ate 4", ">4"), labels = c("Ate 2", ">2
    ate 3", ">3 ate 4", ">4"))
ECD_TREE_C2015$number_children = factor(ECD_TREE_C2015$number_children,
    ordered = TRUE, levels = c("0", "1", "2", "3", "4 ou mais"), labels = c("0", "1", "2", "3",
    "4 ou mais"))
ECD_TREE_C2015$planned_pregnancy = factor(ECD_TREE_C2015$planned_pregnancy,
    ordered = FALSE, levels = c("Planejou", "Sem querer ou mais ou menos"), labels =
    c("Planejou", "Sem querer ou mais ou menos"))
ECD_TREE_C2015$paternal_support = factor(ECD_TREE_C2015$paternal_support,
    ordered = TRUE, levels = c("Low", "Median", "High"), labels = c("Low", "Median",
    "High"))
ECD_TREE_C2015$couple_criticism = factor(ECD_TREE_C2015$couple_criticism, ordered
    = TRUE, levels = c("Low criticism", "Median criticism", "High criticism"), labels =
    c("Low", "Median", "High"))
ECD_TREE_C2015$mother_with_partner =
    factor(ECD_TREE_C2015$mother_with_partner, ordered = FALSE, levels = c("Nao",
    "Sim"), labels = c("Nao", "Sim"))
ECD_TREE_C2015$worked_in_pregnancy =
    factor(ECD_TREE_C2015$worked_in_pregnancy, ordered = FALSE, levels = c("Nao",
    "Sim"), labels = c("Nao", "Sim"))
ECD_TREE_C2015$father_working = factor(ECD_TREE_C2015$father_working, ordered =
    FALSE, levels = c("no", "yes"), labels = c("no", "yes"))
ECD_TREE_C2015$diabetes_pregnancy = factor(ECD_TREE_C2015$diabetes_pregnancy,
    ordered = FALSE, levels = c("Nao", "Sim"), labels = c("Nao", "Sim"))
ECD_TREE_C2015$has_pregnancy = factor(ECD_TREE_C2015$has_pregnancy, ordered
    = FALSE, levels = c("Nao", "Sim"), labels = c("Nao", "Sim"))
ECD_TREE_C2015$maternal_anemia = factor(ECD_TREE_C2015$maternal_anemia,
```

```
        ordered = FALSE, levels = c("Nao", "Sim"), labels = c("Nao", "Sim"))
ECD_TREE_C2015$f_battelle_total_exc_cat =
        factor(ECD_TREE_C2015$f_battelle_total_exc_cat, ordered = FALSE, levels =
        c("Average development", "Suspected developmental delay"), labels = c("Average
        development", "Suspected developmental delay"))
ECD_TREE_C2015$b_IG_grupos = factor(ECD_TREE_C2015$b_IG_grupos, ordered =
        FALSE, levels = c("Very early preterm (18 to <24)", "Early preterm (24 to <34)", "Late
        preterm (34 to <37)", "Early Term (37 0/7 to 38 6/7)", "Full term (39 0/7 to 40 6/7)",
        "Late term (41 0/7 to 41 6/7)", "Post-term (>=42 0/7)"), labels = c("Very early preterm
        (18 to <24)", "Early preterm (24 to <34)", "Late preterm (34 to <37)", "Early Term (37
        0/7 to 38 6/7)", "Full term (39 0/7 to 40 6/7)", "Late term (41 0/7 to 41 6/7)", "Post-term
        (>=42 0/7)"))
ECD_TREE_C2015$bpn = factor(ECD_TREE_C2015$bpn, ordered = FALSE, levels =
        c("2500+", "<2500"), labels = c("2500+", "<2500"))


# Tranforming variavles, characters to integer
ECD_TREE_C2015$maternal_age = as.numeric(ECD_TREE_C2015$maternal_age)
ECD_TREE_C2015$depression_simptons_antenatal =
        as.numeric(ECD_TREE_C2015$depression_simptons_antenatal)
ECD_TREE_C2015$maternal_ACEs = as.numeric(ECD_TREE_C2015$maternal_ACEs)
ECD_TREE_C2015$sispim_gestacao_numeric =
        as.numeric(ECD_TREE_C2015$sispim_gestacao_numeric)


#Generating a tree adjusted for sex and age of the child
set.seed(2)
ctree.model <- ctree(zbattelle_adjidadesex ~ maternal_schooling + paternal_schooling +
        maternal_age +
                        maternal_skin_color + paternal_skin_color +
        neighborhood_violence +
                        family_income_minimum_wage + garbage_accumulated +
        open_sewer +
                        drug_pregnancy + alcohol_pregnancy + smoking_pregnancy +
        smoking_partner +
                        depression_simptons_antenatal +
        depression_simptons_postnatal +
                        density_in_house + number_children + planned_pregnancy +
                        paternal_support + couple_criticism + maternal_ACEs +
        mother_with_partner +
                        worked_in_pregnancy + father_working + diabetes_pregnancy +
        has_pregnancy +
                        maternal_anemia + peson + bpn + b_IG + b_IG_grupos,
        minbucket = 50, alpha = 0.05, data = ECD_TREE_C2015,
                        maxsurrogate = 5)
ctree.model

# Figure
plot(ctree.model, gp = gpar(fontsize = 10))

# Adding nodes
data.predict <- ECD_TREE_C2015
set.seed(2)
```

```
data.predict$node <- predict(ctree.model, newdata = ECD_TREE_C2015)
data.predict <- data.predict %>%
  mutate(node = factor(node))


#Sensitivity Analysis adding gestational age and birthweight and without children receiving
        PIM during pregnancy
ECD_TREE_C2015_sem_pim <-
        ECD_TREE_C2015[ECD_TREE_C2015$sispim_gestacao_numeric!=1, ]


#Generating a tree ajusted for sex and age of the child
set.seed(2)
ctree.model <- ctree(zbattelle_adjidadesex ~ maternal_schooling + paternal_schooling +
        maternal_age +
                maternal_skin_color + paternal_skin_color + neighborhood_violence +
                family_income_minimum_wage + garbage_accumulated + open_sewer +
                drug_pregnancy + alcohol_pregnancy + smoking_pregnancy +
        smoking_partner +
                depression_simptons_antenatal + depression_simptons_postnatal +
                density_in_house + number_children + planned_pregnancy +
                paternal_support + couple_criticism + maternal_ACEs + mother_with_partner
        +
                worked_in_pregnancy + father_working + diabetes_pregnancy +
        has_pregnancy +
                maternal_anemia + peson + bpn + b_IG + b_IG_grupos, minbucket = 50,
        alpha = 0.05, data = ECD_TREE_C2015,
              maxsurrogate = 5)
ctree.model

# Figure
plot(ctree.model, gp = gpar(fontsize = 10))

# Adding nodes
data.predict <- ECD_TREE_C2015
set.seed(2)
data.predict$node <- predict(ctree.model, newdata = ECD_TREE_C2015)
data.predict <- data.predict %>%
  mutate(node = factor(node))
```

Code used in STATA version 15.1

```
//Supplemental Table 1
tab maternal_skin_color,m
tab maternal_skin_color if z_battelle!=. & Idade_meses!=.,m
tab maternal_skin_color if z_battelle!=. & Idade_meses!=.
tab paternal_skin_color,m
tab paternal_skin_color if z_battelle!=. & Idade_meses!=.,m
tab paternal_skin_color if z_battelle!=. & Idade_meses!=.
tab neighborhood_violence,m
```

```
tab neighborhood_violence if z_battelle!=. & Idade_meses!=.,m
tab neighborhood_violence if z_battelle!=. & Idade_meses!=.
tab garbage_accumulated,m
tab garbage_accumulated if z_battelle!=. & Idade_meses!=.,m
tab garbage_accumulated if z_battelle!=. & Idade_meses!=.
tab open_sewer,m
tab open_sewer if z_battelle!=. & Idade_meses!=.,m
tab open_sewer if z_battelle!=. & Idade_meses!=.
tab drug_pregnancy,m
tab drug_pregnancy if z_battelle!=. & Idade_meses!=.,m
tab drug_pregnancy if z_battelle!=. & Idade_meses!=.
tab alcohol_pregnancy ,m
tab alcohol_pregnancy if z_battelle!=. & Idade_meses!=.,m
tab alcohol_pregnancy if z_battelle!=. & Idade_meses!=.
tab smoking_pregnancy ,m
tab smoking_pregnancy if z_battelle!=. & Idade_meses!=.,m
tab smoking_pregnancy if z_battelle!=. & Idade_meses!=.
tab smoking_partner ,m
tab smoking_partner if z_battelle!=. & Idade_meses!=.,m
tab smoking_partner if z_battelle!=. & Idade_meses!=.
tab depr_simpt_antenat_dic,m
tab depr_simpt_antenat_dic if z_battelle!=. & Idade_meses!=.,m
tab depr_simpt_antenat_dic if z_battelle!=. & Idade_meses!=.
tab depr_simpt_postnat_dic ,m
tab depr_simpt_postnat_dic if z_battelle!=. & Idade_meses!=.,m
tab depr_simpt_postnat_dic if z_battelle!=. & Idade_meses!=.
tab density_in_house,m
tab density_in_house if z_battelle!=. & Idade_meses!=.,m
tab density_in_house if z_battelle!=. & Idade_meses!=.
tab number_children ,m
tab number_children if z_battelle!=. & Idade_meses!=.,m
tab number_children if z_battelle!=. & Idade_meses!=.
tab planned_pregnancy ,m
tab planned_pregnancy if z_battelle!=. & Idade_meses!=.,m
tab planned_pregnancy if z_battelle!=. & Idade_meses!=.
tab paternal_support ,m
tab paternal_support if z_battelle!=. & Idade_meses!=.,m
tab paternal_support if z_battelle!=. & Idade_meses!=.
tab couple_criticism ,m
tab couple_criticism if z_battelle!=. & Idade_meses!=.,m
tab couple_criticism if z_battelle!=. & Idade_meses!=.
tab mother_with_partner ,m
tab mother_with_partner if z_battelle!=. & Idade_meses!=.,m
tab mother_with_partner if z_battelle!=. & Idade_meses!=.
tab worked_in_pregnancy ,m
tab worked_in_pregnancy if z_battelle!=. & Idade_meses!=.,m
tab worked_in_pregnancy if z_battelle!=. & Idade_meses!=.
tab father_working ,m
tab father_working if z_battelle!=. & Idade_meses!=.,m
tab father_working if z_battelle!=. & Idade_meses!=.
tab diabetes_pregnancy ,m
tab diabetes_pregnancy if z_battelle!=. & Idade_meses!=.,m
tab diabetes_pregnancy if z_battelle!=. & Idade_meses!=.
```

```
tab has_pregnancy ,m
tab has_pregnancy if z_battelle!=. & Idade_meses!=.,m
tab has_pregnancy if z_battelle!=. & Idade_meses!=.
tab maternal_anemia ,m
tab maternal_anemia if z_battelle!=. & Idade_meses!=.,m
tab maternal_anemia if z_battelle!=. & Idade_meses!=.
sum maternal_age
sum maternal_age if z_battelle!=. & Idade_meses!=.
sum maternal_schooling
sum maternal_schooling if z_battelle!=. & Idade_meses!=.
sum paternal_schooling
sum paternal_schooling if z_battelle!=. & Idade_meses!=.
sum family_income_minimum_wage
sum family_income_minimum_wage if z_battelle!=. & Idade_meses!=.
sum maternal_ACEs
sum maternal_ACEs if z_battelle!=. & Idade_meses!=.


//Supplemental Table 2
regress z_battelle i.maternal_skin_color if Idade_meses!=.
regress z_battelle i.paternal_skin_color if Idade_meses!=.
regress z_battelle i.neighborhood_violence if Idade_meses!=.
regress z_battelle garbage_accumulated if Idade_meses!=.
regress z_battelle open_sewer if Idade_meses!=.
regress z_battelle drug_pregnancy if Idade_meses!=.
regress z_battelle alcohol_pregnancy if Idade_meses!=.
regress z_battelle smoking_pregnancy if Idade_meses!=.
regress z_battelle smoking_partner if Idade_meses!=.
regress z_battelle depr_simpt_antenat_dic if Idade_meses!=.
regress z_battelle depr_simpt_postnat_dic if Idade_meses!=.
regress z_battelle i.density_in_house if Idade_meses!=.
regress z_battelle i.number_children if Idade_meses!=.
regress z_battelle planned_pregnancy if Idade_meses!=.
regress z_battelle i.paternal_support if Idade_meses!=.
regress z_battelle i.couple_criticism if Idade_meses!=.
regress z_battelle mother_with_partner if Idade_meses!=.
regress z_battelle worked_in_pregnancy if Idade_meses!=.
regress z_battelle father_working if Idade_meses!=.
regress z_battelle diabetes_pregnancy if Idade_meses!=.
regress z_battelle has_pregnancy if Idade_meses!=.
regress z_battelle maternal_anemia if Idade_meses!=.
regress z_battelle maternal_age if Idade_meses!=.
regress z_battelle maternal_schooling if Idade_meses!=.
regress z_battelle paternal_schooling if Idade_meses!=.
regress z_battelle family_income_minimum_wage if Idade_meses!=.
regress z_battelle maternal_ACEs if Idade_meses!=.


//Examining correlation among predictors
pwcorr maternal_schooling paternal_schooling maternal_age maternal_skin_color
        paternal_skin_color neighborhood_violence family_income_minimum_wage
        garbage_accumulated open_sewer drug_pregnancy alcohol_pregnancy
        smoking_pregnancy smoking_partner depression_simptons_antenatal
```

```
    depression_simptons_postnatal density_in_house number_children
    planned_pregnancy paternal_support couple_criticism maternal_ACEs
    mother_with_partner worked_in_pregnancy father_working diabetes_pregnancy
    has_pregnancy maternal_anemia


//Table 1 - linear regression
regress z_battelle maternal_schooling paternal_schooling i.family_income_min_wage_quint
    mat_skinc_x_depr_symp alc_preg_x_mat_aces Idade_meses sex if
    analytic_sample_regress!=.
testparm i.family_income_min_wage_quint

regress z_battelle maternal_schooling paternal_schooling i.family_income_min_wage_quint
    mat_skinc_x_depr_symp alc_preg_x_mat_aces Idade_meses sex if
    analytic_sample_regress!=. & sispim_gestacao_numeric!=1
testparm i.family_income_min_wage_quint

/Adjusted R-squared
regress zbattelle_adjidadesex maternal_schooling paternal_schooling
    i.family_income_min_wage_quint mat_skinc_x_depr_symp alc_preg_x_mat_aces if
    analytic_sample_regress!=.


//Table 2 and supplemental table 3- PCA
pca maternal_age_dic maternal_schooling paternal_schooling
    family_income_min_wage_quint maternal_skin_color_dic paternal_skin_color_dic
    number_children smoking_pregnancy alcohol_pregnancy maternal_ACEs
    depr_simpt_postnat_3cat, comp(4)
estat kmo
screeplot, mean ci
predict pc1 pc2 pc3 pc4
regress z_battelle pc1 pc2 pc3 pc4 sex Idade_meses
/Adjusted R-squared
regress zbattelle_adjidadesex pc1 pc2 pc3 pc4


//Examining association between Maternal schooling, Paternal schooling and Standardized
    BDI
fp <maternal_schooling>, scale: regress z_battelle <maternal_schooling>
estat ic
regress z_battelle maternal_schooling
estat ic
fracpoly, deg(2)compare log: reg z_battelle maternal_schooling
sc z_battelle maternal_schooling || lowess z_battelle maternal_schooling
sc z_battelle maternal_schooling || lfit z_battelle maternal_schooling
regress z_battelle maternal_schooling
predict res,residuals
sc res maternal_schooling || lowess res maternal_schooling, yline(0)

fp <paternal_schooling>, scale: regress z_battelle <paternal_schooling>
estat ic
regress z_battelle paternal_schooling
estat ic
```

```
fracpoly, deg(2)compare log: reg z_battelle paternal_schooling
sc z_battelle paternal_schooling || lowess z_battelle paternal_schooling
sc z_battelle paternal_schooling || lfit z_battelle paternal_schooling
regress z_battelle paternal_schooling
predict res,residuals
sc res paternal_schooling || lowess res paternal_schooling, yline(0)

//Generating fitted values to be used as a parameter for AUC e PIM coverage and focus
regress zbattelle_adjidadesex maternal_schooling paternal_schooling
predict fitted_ECDADJ_parentalschool
xtile fitted_ECDADJ_parentalschoolDEC = fitted_ECDADJ_parentalschool, nq(10)

//ROC curve, AUC, sensitivity and specificity
roctab f_battelle_total_exc_cat fitted_ECDADJ_parentalschool, binomial detail graph
        plotopts(recast(connected))
recode fitted_ECDADJ_parentalschool min/-.0197698=1 -.0197697/max=0, gen (
        fitted_ECDADJ_parentalschoolDIC )
whelp diagt
diagt f_battelle_total_exc_cat fitted_ECDADJ_parentalschoolDIC
logistic f_battelle_total_exc_cat fitted_ECDADJ_parentalschool
lroc

//Sensitivity analysis excluding 142 PIM children: Fitted BDI, ROC curve, AUC, sensitivity
        and specificity
regress zbattelle_adjidadesex maternal_schooling paternal_schooling if sispim_gestacao!=1
predict fitted_ECDADJ_parentalschoolSENS if sispim_gestacao!=1
roctab f_battelle_total_exc_cat fitted_ECDADJ_parentalschoolSENS , binomial detail graph
        plotopts(recast(connected))
recode fitted_ECDADJ_parentalschoolSENS min/-.0197698=1 -.0197699/max=0, gen (
        fitted_ECDADJ_DIC)
diagt f_battelle_total_exc_cat fitted_ECDADJ_DIC


//Coverage and focus of PIM
/Figure 4
recode sispim_gestacao_numeric 0=. if pim_itt_final==.
//Coverage
tab fitted_ECD_parentalschool_dec sispim_gestacao_numeric , chi row
graph bar sispim_gestacao_numeric, over(fitted_ECD_parentalschool_dec)
//Focus
tab fitted_ECD_parentalschool_dec sispim_gestacao_numeric , chi col
//Focus vs duration
recode sispim_permanencia_cat 0/1=0 2/4=1, gen ( sispim_permanencia_12m)
tab fitted_ECD_parentalschool_dec sispim_permanencia_12m , chi col
tab fitted_ECD_parentalschool_dec sispim_permanencia_12m if
        sispim_gestacao_numeric==1 , chi col
/Supplemental figure 9
recode sispim_afterbirth_numeric 0=. if pim_itt_final==.
//Coverage
tab fitted_ECD_parentalschool_dec sispim_afterbirth_numeric , chi row
//Focus
tab fitted_ECD_parentalschool_dec sispim_afterbirth_numeric , chi col
//Focus vs duration
```

```
tab fitted_ECD_parentalschool_dec sispim_permanencia_12m if
    sispim_afterbirth_numeric==1 , chi col


///External validation with 2004 cohort
//Examining association between Maternal schooling, Paternal schooling and Standardized
    BDI
fp <maternal_schooling>, scale: regress z_battelle <maternal_schooling>
estat ic
regress z_battelle maternal_schooling
estat ic
fracpoly, deg(2)compare log: reg z_battelle maternal_schooling
sc z_battelle maternal_schooling || lowess z_battelle maternal_schooling
sc z_battelle maternal_schooling || lfit z_battelle maternal_schooling
regress z_battelle maternal_schooling
predict res,residuals
sc res maternal_schooling || lowess res maternal_schooling, yline(0)

fp <paternal_schooling>, scale: regress z_battelle <paternal_schooling>
estat ic
regress z_battelle paternal_schooling
estat ic
fracpoly, deg(2)compare log: reg z_battelle paternal_schooling
sc z_battelle paternal_schooling || lowess z_battelle paternal_schooling
sc z_battelle paternal_schooling || lfit z_battelle paternal_schooling
regress z_battelle paternal_schooling
predict res,residuals
sc res paternal_schooling || lowess res paternal_schooling, yline(0)

//Generating fitted values to be used as a parameter for AUC e PIM coverage and focus
regress z_battelle sex Idade_meses
predict zbattelle_adjidadesex,residuals
regress zbattelle_adjidadesex maternal_schooling paternal_schooling
predict fitted_ECDADJ_parentalschool
//ROC curve, AUC, sensitivity and specificity
roctab f_battelle_total_exc_cat fitted_ECDADJ_parentalschool, binomial detail graph
    plotopts(recast(connected))
recode fitted_ECDADJ_parentalschool min/-.0197698=1 -.0197697/max=0, gen (
    fitted_ECDADJ_DIC )
whelp diagt
diagt f_battelle_total_exc_cat fitted_ECDADJ_DIC


///Supplemental table 4
recode fitted_ECDADJ_parentalschool min/-.0221936=1 -.0221935/max=0, gen (
    fitted_ECDADJ_parentalschoolDICB )
diagt f_battelle_total_exc_cat fitted_ECDADJ_parentalschoolDICB
recode fitted_ECDADJ_parentalschool min/-.0246175=1 -.0246174/max=0, gen (
    fitted_ECDADJ_parentalschoolDICC )
diagt f_battelle_total_exc_cat fitted_ECDADJ_parentalschoolDICC
recode fitted_ECDADJ_parentalschool min/-.0288267=1 -.0288266/max=0, gen (
    fitted_ECDADJ_parentalschoolDICD )
diagt f_battelle_total_exc_cat fitted_ECDADJ_parentalschoolDICD
```

```
recode fitted_ECDADJ_parentalschool min/-.0131367=1 -.0131366/max=0, gen (
        fitted_ECDADJ_parentalschoolDICE )
diagt f_battelle_total_exc_cat fitted_ECDADJ_parentalschoolDICE
recode fitted_ECDADJ_parentalschool min/-.0107129=1 -.0107128/max=0, gen (
        fitted_ECDADJ_parentalschoolDICF )
diagt f_battelle_total_exc_cat fitted_ECDADJ_parentalschoolDICF
recode fitted_ECDADJ_parentalschool min/-.008289=1 -.008288/max=0, gen (
        fitted_ECDADJ_parentalschoolDICG )
diagt f_battelle_total_exc_cat fitted_ECDADJ_parentalschoolDICG



///Post-hoc analysis with perinatal predictors
//Supplemental table 7 - linear regression
regress z_battelle maternal_schooling paternal_schooling i.family_income_min_wage_quint
        mat_skinc_x_depr_symp alc_preg_x_mat_aces b_IG peson Idade_meses sex if
        analytic_sample_regress!=.
testparm i.family_income_min_wage_quint

regress z_battelle maternal_schooling paternal_schooling i.family_income_min_wage_quint
        mat_skinc_x_depr_symp alc_preg_x_mat_aces b_IG peson Idade_meses sex if
        analytic_sample_regress!=. & sispim_gestacao_numeric!=1
testparm i.family_income_min_wage_quint

//Adjusted R-squared
regress zbattelle_adjidadesex maternal_schooling paternal_schooling
        i.family_income_min_wage_quint mat_skinc_x_depr_symp alc_preg_x_mat_aces
        b_IG peson if analytic_sample_regress!=.


//Supplemental table 8 and supplemental table 9 - PCA
pca maternal_age_dic maternal_schooling paternal_schooling
        family_income_min_wage_quint maternal_skin_color_dic paternal_skin_color_dic
        number_children smoking_pregnancy alcohol_pregnancy maternal_ACEs
        depr_simpt_postnat_3cat b_IG peson, comp(5)
estat kmo
screeplot, mean ci
predict pc1 pc2 pc3 pc4 pc5
regress z_battelle pc1 pc2 pc3 pc4 pc5 sex Idade_meses
/Adjusted R-squared
regress zbattelle_adjidadesex pc1 pc2 pc3 pc4 pc5
```

REFERENCES

1 Newborg J, Stock J, Wnek L. Battelle Developmental Inventory. Itasca: Riverside; 1988.

2 Barros AJD, Matijasevich A, Santos IS, Halpern R. Child development in a birth cohort: Effect of child stimulation is stronger in less educated mothers. Int J Epidemiol. 2010;39(1):285–94.

3 Hothorn T, Hornik K, Zeileis A. Unbiased recursive partitioning: A conditional inference framework. J Comput Graph Stat. 2006;15(3):651–74.

4 Hothorn T, Seibold H, Zeileis A. Package “partykit” - A Toolkit for Recursive Partytioning. R package. 2023. Available: https://cran.r-project.org/web/packages/partykit/partykit.pdf. Accessed: 15 May 2023.

5 Venkatasubramaniam A, Wolfson J, Mitchell N, Barnes T, Jaka M, French S. Decision trees in epidemiological research. Emerg Themes Epidemiol. 2017;14(1):1–12.
